# Supplementary material for: Ionic and Non-Ionic Counterparts Based on Bis(Uracilyl)Alkane Moiety with Highest Selectivity Towards Acetylcholinesterase for Protection Against Organophosphate Poisoning and Treating Alzheimer’s Disease
Source: Int J Mol Sci. 2025 Apr 16;26(8):3759. doi: 10.3390/ijms26083759 (PMC12027946; doi:10.3390/ijms26083759)
Supplement: Supplementary file 1 [file ijms-26-03759-s001.zip › ijms-3553861-supplementary.pdf]

Supplementary Information

Ionic and non-ionic counterparts based on bis(uracilyl)alkane moiety with highest selectivity towards acetylcholinesterase for protection against organophosphate poisoning and treating Alzheimer's disease

Irina V. Zueva [a], Liliya F. Saifina [a], Liliya M. Gubaidullina [a], Marina M. Shulaeva [a], Alexandra D. Kharlamova [a], Oksana A. Lenina [a], Grigory P. Belyaev [a], Albina Ziganshina [a], Shan Gao [b], Wenjian Tang [b,*], Vyacheslav E. Semenov [a,*], Konstantin A. Petrov [a,c]

[a] *Arbuzov Institute of Organic and Physical Chemistry, FRC Kazan Scientific Center of RAS Arbuzov str., 8, Kazan, 420088, Russian Federation*

[b] *School of Pharmacy, Anhui Medical University, Hefei, 230032, China*

[c] *Kazan Federal University, 18 Kremlyovskaya str, Kazan, 420008, Russia*

Table of contents **Pages**

Table of contents……S1
Spectra of the compounds synthesized……S2
NMR experiments details……S2
MS experiments details……S2
Figure S1. MALDI-TOF mass spectrum of **2a** ……S3
Figure S2. 1D ^{1}H NMR spectrum of **2a** in $CDCl_3$ at T = 303 K……S4
Figure S3. 1D ^{13}C NMR spectrum of **2a** in $CDCl_3$ at T = 303 K ……S4
Figure S4. MALDI-TOF mass spectrum of **2b** ……S5
Figure S5. 1D ^{1}H NMR spectrum of **2b** in $CDCl_3$ at T = 303 K……S6
Figure S6. 1D ^{13}C NMR spectrum of **2b** in $CDCl_3$ at T = 303 K ……S6
Figure S7. MALDI-TOF mass spectrum of **2c** ……S7
Figure S8. 1D ^{1}H NMR spectrum of **2c** in $CDCl_3$ at T = 303 K……S8
Figure S9. 1D ^{13}C NMR spectrum of **2c** in $CDCl_3$ at T = 303 K ……S8
Figure S10. MALDI-TOF mass spectrum of **2d**……S9
Figure S11. 1D ^{1}H NMR spectrum of **2d** in $CDCl_3$ at T = 303 K……S10
Figure S12. 1D ^{13}C NMR spectrum of **2d** in $CDCl_3$ at T = 303 K ……S10
Figure S13. ESI mass spectrum of **10a**……S11
Figure S14. 1D ^{1}H NMR spectrum of **10a** in $CDCl_3$ at T = 303 K……S12
Figure S15. 1D ^{13}C NMR spectrum of **10a** in $CDCl_3$ at T = 303 K ……S12
Figure S16. MALDI-TOF mass spectrum of **10b**……S13
Figure S17. 1D ^{1}H NMR spectrum of **10b** in $CDCl_3$ at T = 303 K……S14
Figure S18. 1D ^{13}C NMR spectrum of **10b** in $CDCl_3$ at T = 303 K ……S14
Figure S19. 1D ^{1}H NMR spectrum of **10c** in $CDCl_3$ at T = 303 K……S15
Figure S20. 1D ^{13}C NMR spectrum of **10c** in $CDCl_3$ at T = 303 K ……S15

Figure S21. MALDI-TOF mass spectrum of **10d.**………………………………………...............................S16
Figure S22. 1D ^{1}H NMR spectrum of **10d** in $CDCl_3$ at T = 303 K...……………………...……….……..S17
Figure S23. 1D ^{13}C NMR spectrum of **10d** in $CDCl_3$ at T = 303 K ……………...……………...…......S17
Figure S24. MALDI-TOF mass spectrum of **3a,.**………………………………………...............................S18
Figure S25. 1D ^{1}H NMR spectrum of **3a** in $CDCl_3$ at T = 303 K..……..………………...……..………...S19
Figure S26. 1D ^{13}C NMR spectrum of **3a** in $CDCl_3$ at T = 303 K ………………..…………….…..……S19
Figure S27. MALDI-TOF mass spectrum of **3b**...………………………………………...............................S20
Figure S28. 1D ^{1}H NMR spectrum of **3b** in $CDCl_3$ at T = 303 K..……..………………..……...………..S21
Figure S29. 1D ^{13}C NMR spectrum of **3b** in $CDCl_3$ at T = 303 K ……………...………………...…...S21
Figure S30. MALDI-TOF mass spectrum of **3c**...………………………………………...............................S22
Figure S31. 1D ^{1}H NMR spectrum of **3c** in $CDCl_3$ at T = 303 K..……..………………...……..………..S23
Figure S32. 1D ^{13}C NMR spectrum of **3c** in $CDCl_3$ at T = 303 K ………………..…………….…..……S23
Figure S33. MALDI-TOF mass spectrum of **3d**...………………………………………...............................S24
Figure S34. 1D ^{1}H NMR spectrum of **3d** in $CDCl_3$ at T = 303 K..……..………………...……..………S25
Figure S35. 1D ^{13}C NMR spectrum of **3d** in $CDCl_3$ at T = 303 K ………………..…………….…..……S25

NMR spectra of the compounds synthesized

NMR experiments details

All NMR experiments were performed with 400.1 MHz for ^{1}H NMR, and 100.6 for ^{13}C NMR spectrometer equipped with 5 mm diameter gradient inverse broad band probehead and a pulsed gradient unit capable of producing magnetic field pulse gradients in the *z*-direction of 53.5 G·cm^{-1}. NMR experiments were carried out at 303 K. Chemical shifts (δ in ppm) were referenced to the $CDCl_3$ (δ = 7.26 ppm for ^{1}H and 77.0 ppm for ^{13}C NMR)).

MS experiments details

MALDI-TOF mass spectra were recorded in a positive and negative ion mode on a Bruker ULTRAFLEX III mass spectrometer (Bruker Daltonic GmbH, Bremen, Germany) using *p*-nitroaniline as a matrix for 10^{-3} mg/ml solutions in MeOH. A Nd:YAG laser (λ = 355 nm, repetition rate 100 Hz) was used. The mass spectrum was obtained with an accelerating voltage of 25 kV and an ion extraction delay time of 30 ns. The resulting mass spectrum was formed due to multiple laser irradiation of the crystal (50 shots). The metal target MTP AnchorChipTM was used. Portions (0.5 μl) of a 1% matrix solution in acetonitrile and sample solution were consecutively applied onto the target and evaporated. The polyethylene glycol was used to calibrate the mass scale of the device. The data was obtained using the FlexControl program and processed using the FlexAnalysis 3.0 program (Bruker Daltonik GmbH, Germany).

The ESI MS measurements were performed using an AmazonX ion trap mass spectrometer (Bruker Daltonic GmbH, Germany) in positive (and/or negative) mode in the mass range of 70–3000. The capillary voltage was −3500 V, nitrogen drying gas – 10 L·min^{-1}, desolvation temperature – 250 °C. An methanol/water solution (70:30) was used as a mobile phase at a flow rate of 0.2 mL/min by binary pump (Agilent 1260 chromatograph, USA). The sample was dissolved in methanol to a concentration of 10-6 g•L^{-1}. The instrument was calibrated with a tuning mixture (Agilent G2431A, USA). For instrument control and data acquiring the TrapControl 7.0 software (Bruker Daltonik GmbH, Germany) was used. Data processing was performed by DataAnalysis 4.0 SP4 software (Bruker Daltonik GmbH, Germany).

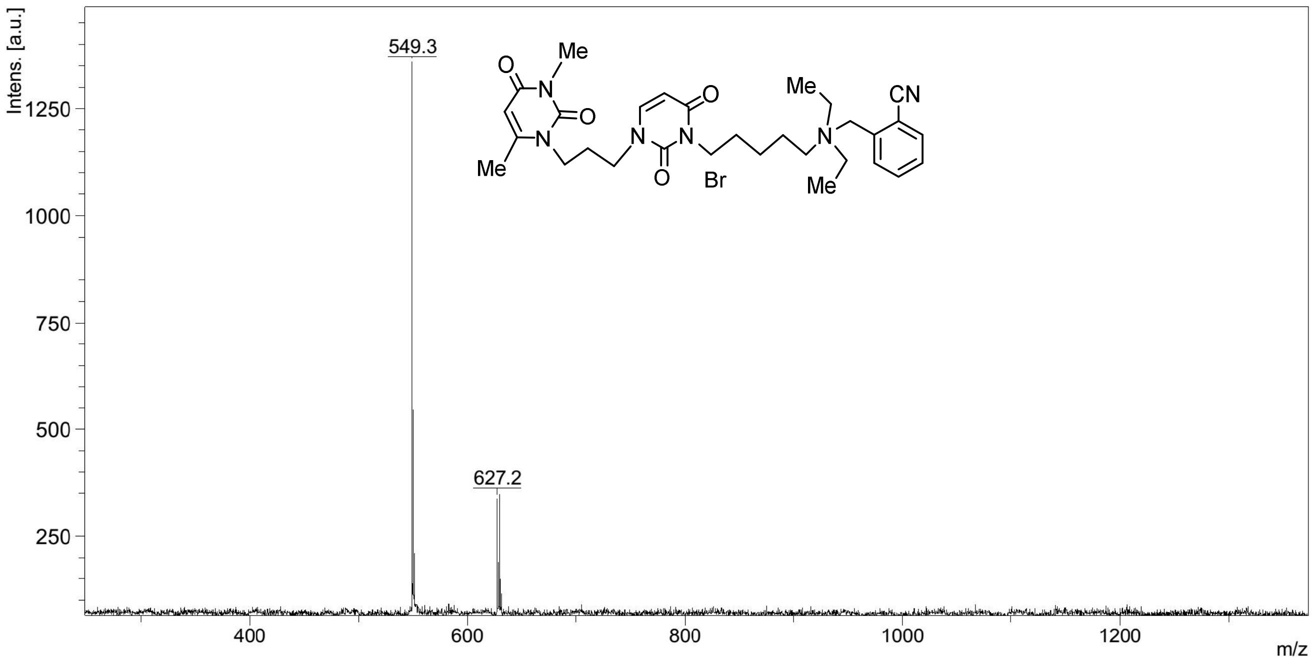

Figure S1. MALDI-TOF mass spectrum of **2a**.

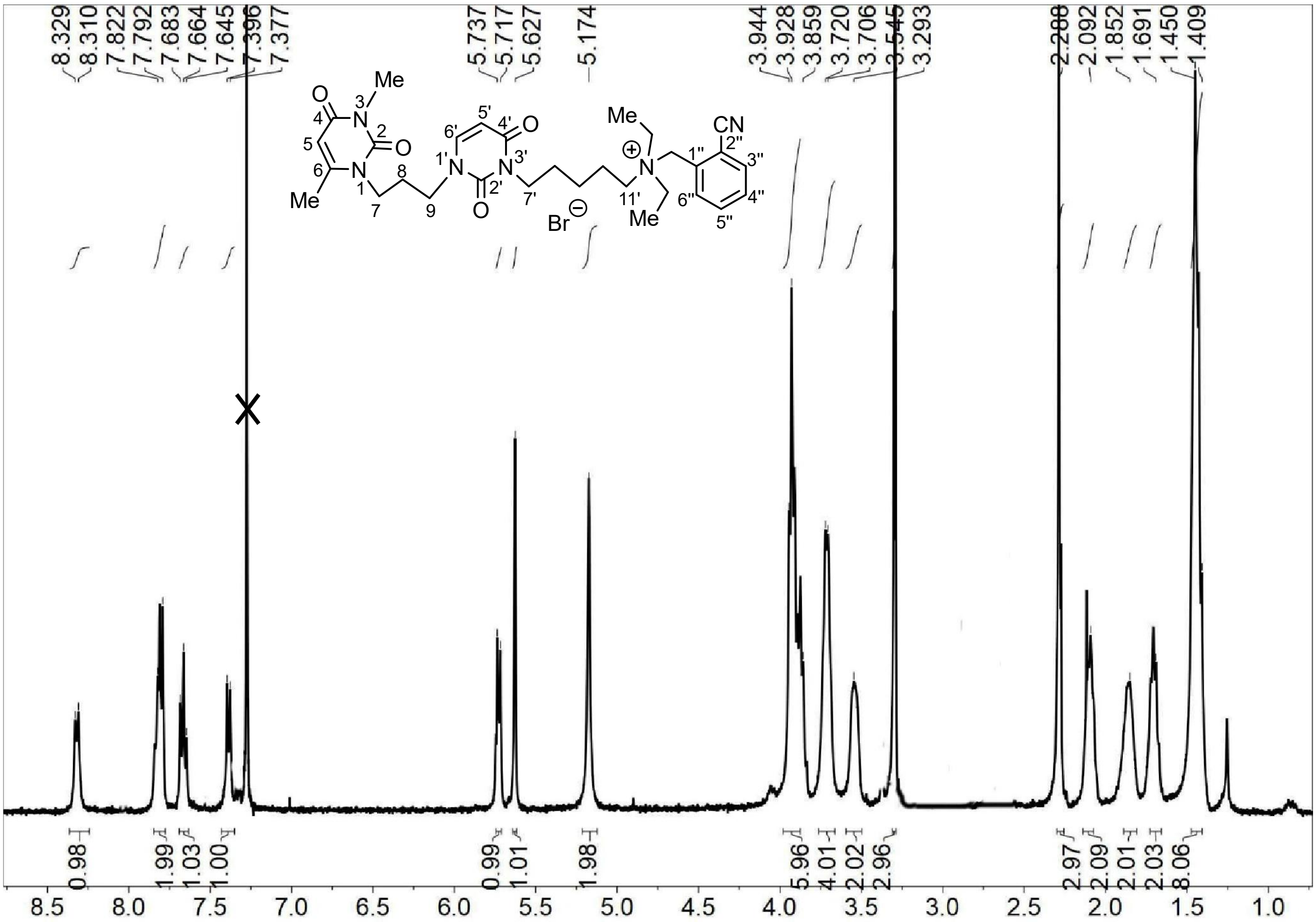

Figure S2. 1D ^1H NMR spectrum of **2a** in $CDCl_3$ (400 MHz) at T = 303 K. x - residual solvent peak.

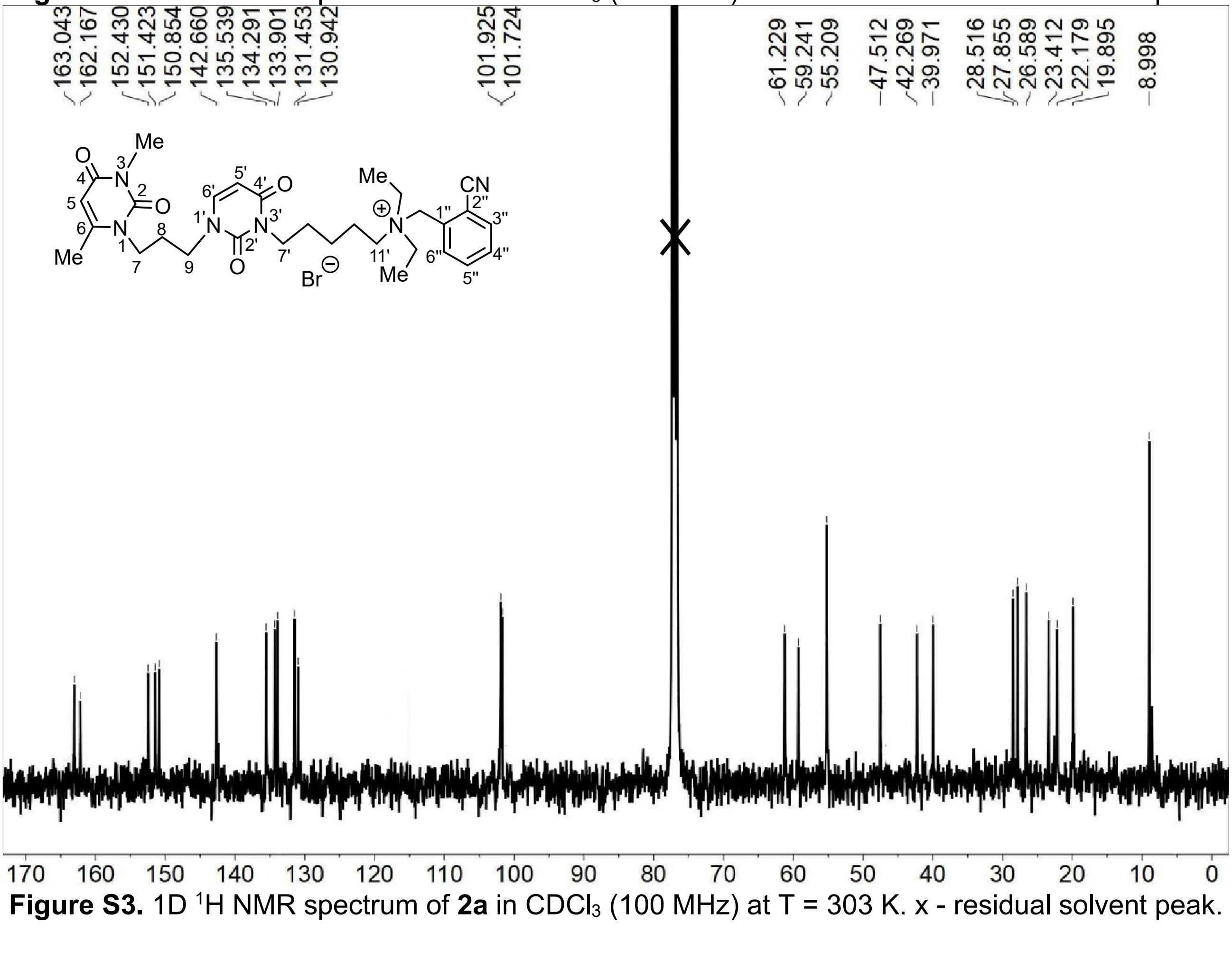

Figure S3. 1D ^1H NMR spectrum of **2a** in $CDCl_3$ (100 MHz) at T = 303 K. x - residual solvent peak.

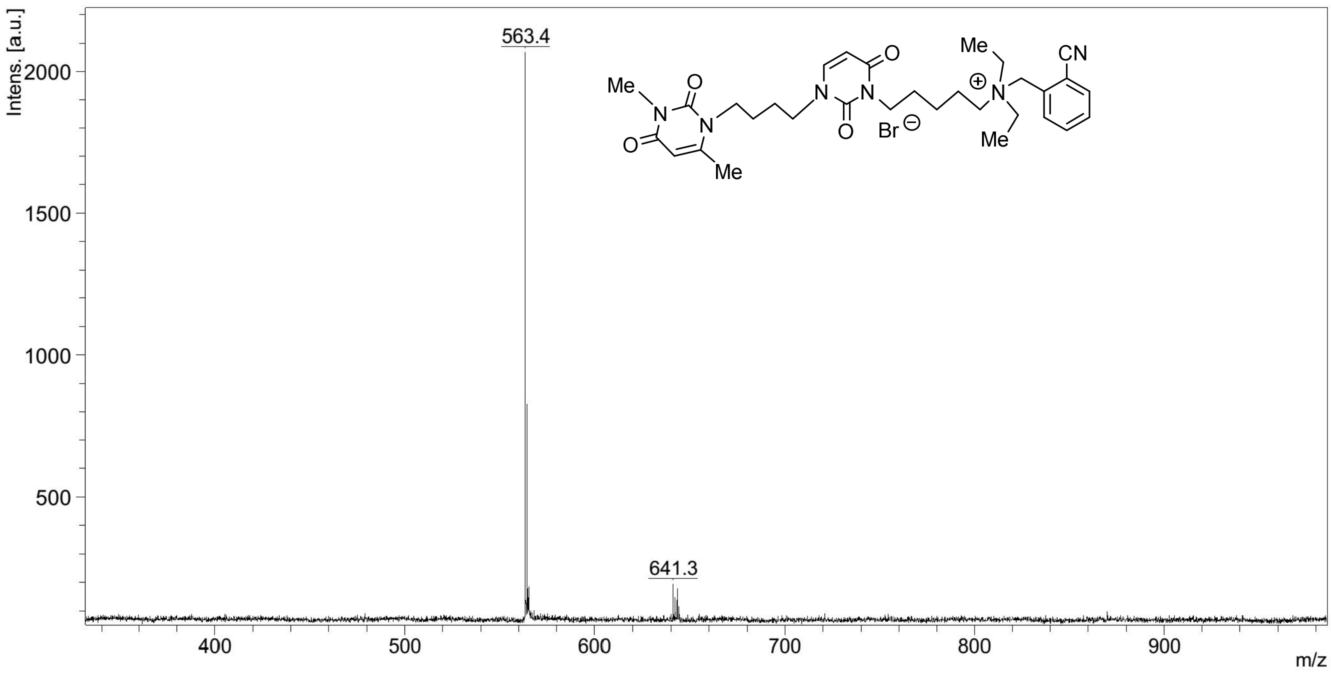

Figure S4. MALDI-TOF mass spectrum of **2b**.

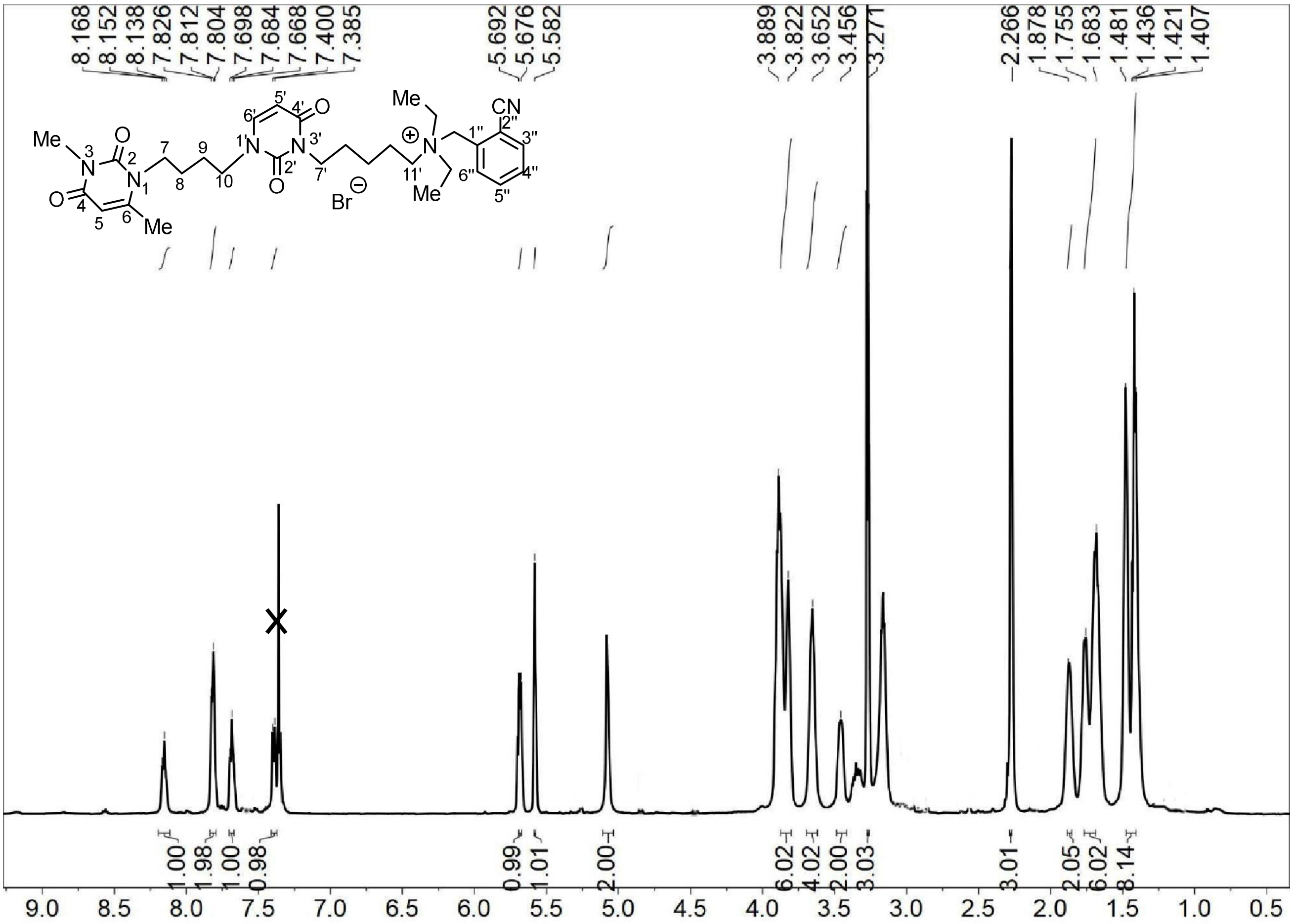

Figure S5. 1D ^{1}H NMR spectrum of **2b** in $CDCl_3$ (400 MHz) at T = 303 K. x - residual solvent peak.

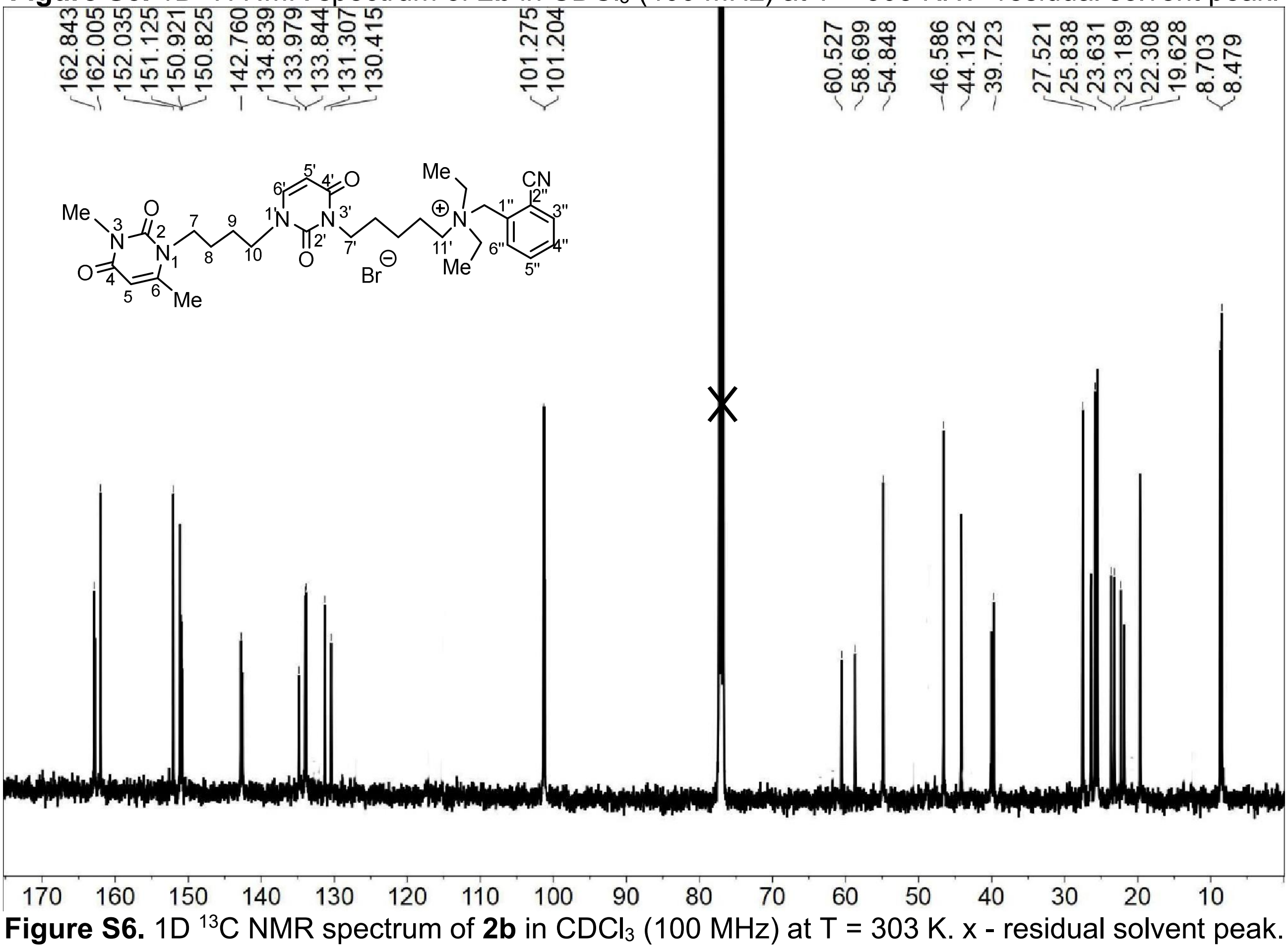

Figure S6. 1D ^{13}C NMR spectrum of **2b** in $CDCl_3$ (100 MHz) at T = 303 K. x - residual solvent peak.

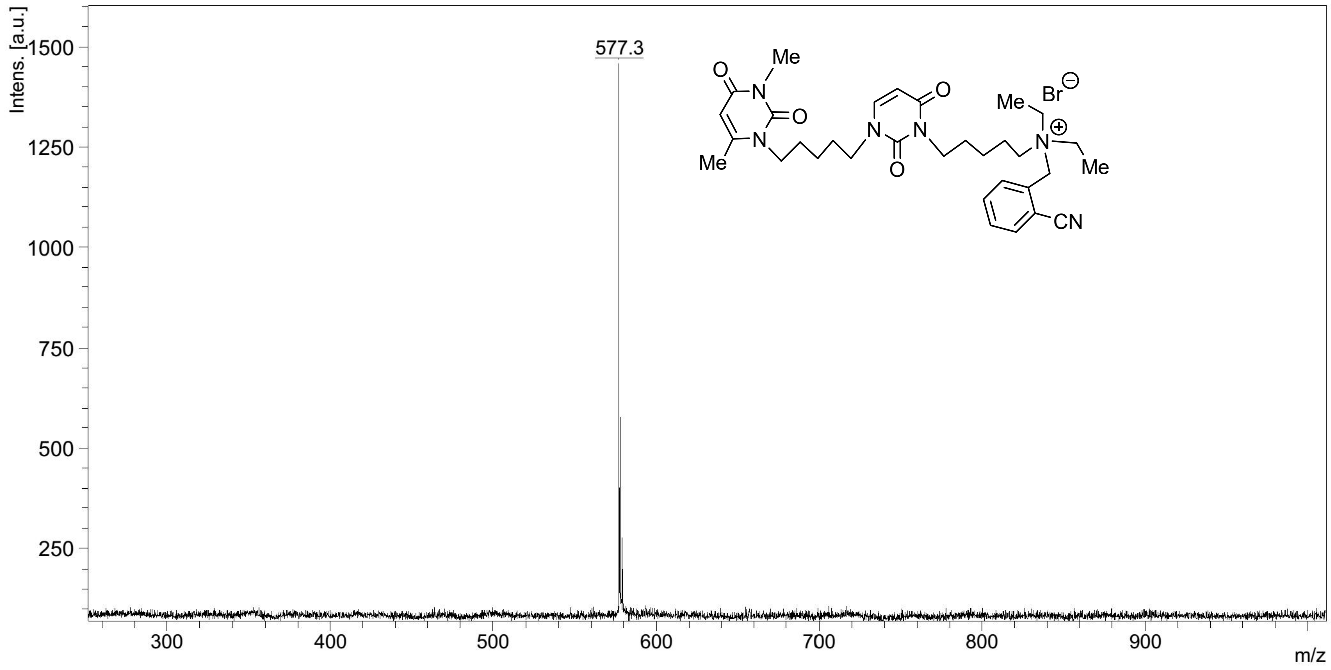

Figure S7. MALDI-TOF mass spectrum of **2c**.

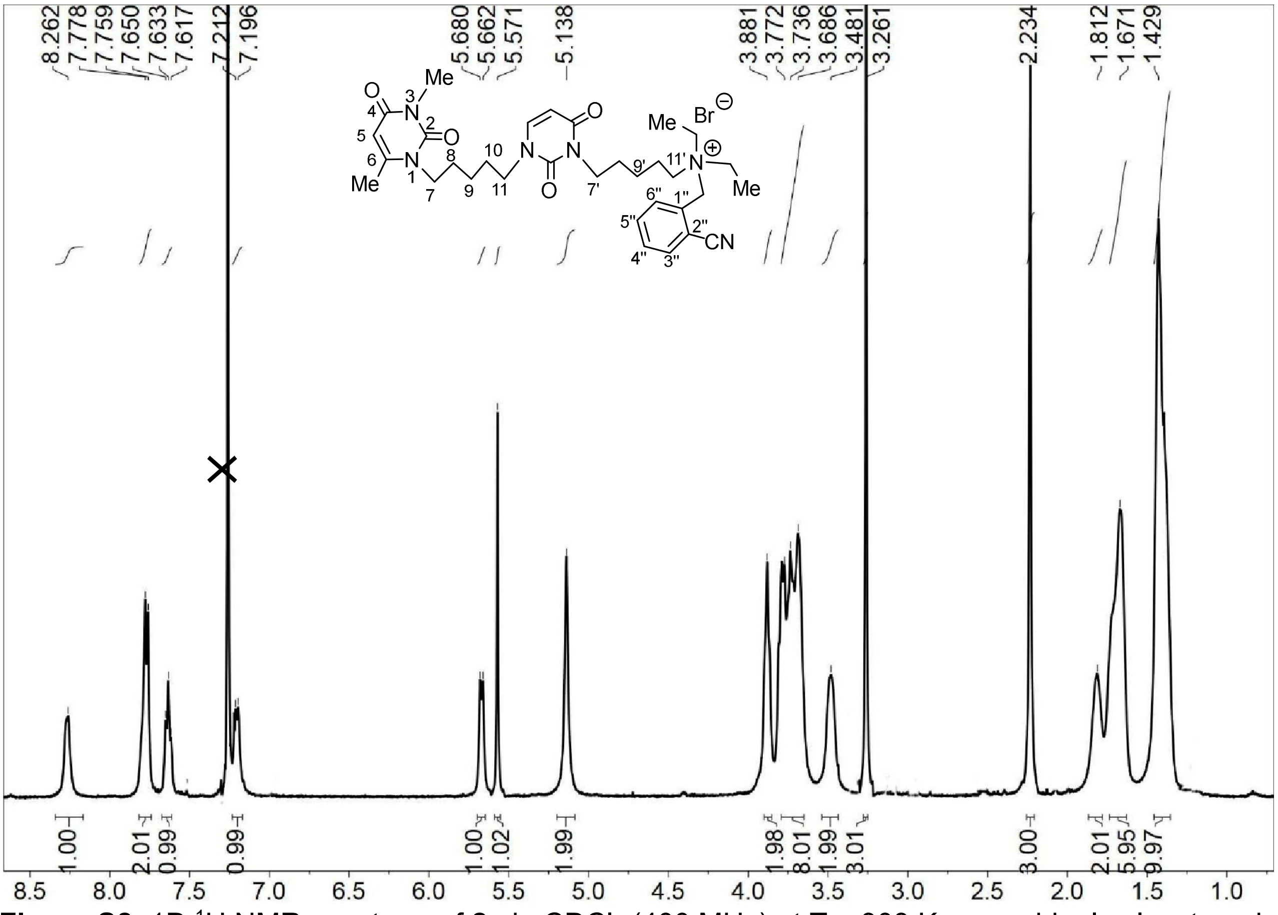

Figure S8. 1D ^1H NMR spectrum of **2c** in $CDCl_3$ (400 MHz) at T = 303 K. x - residual solvent peak.

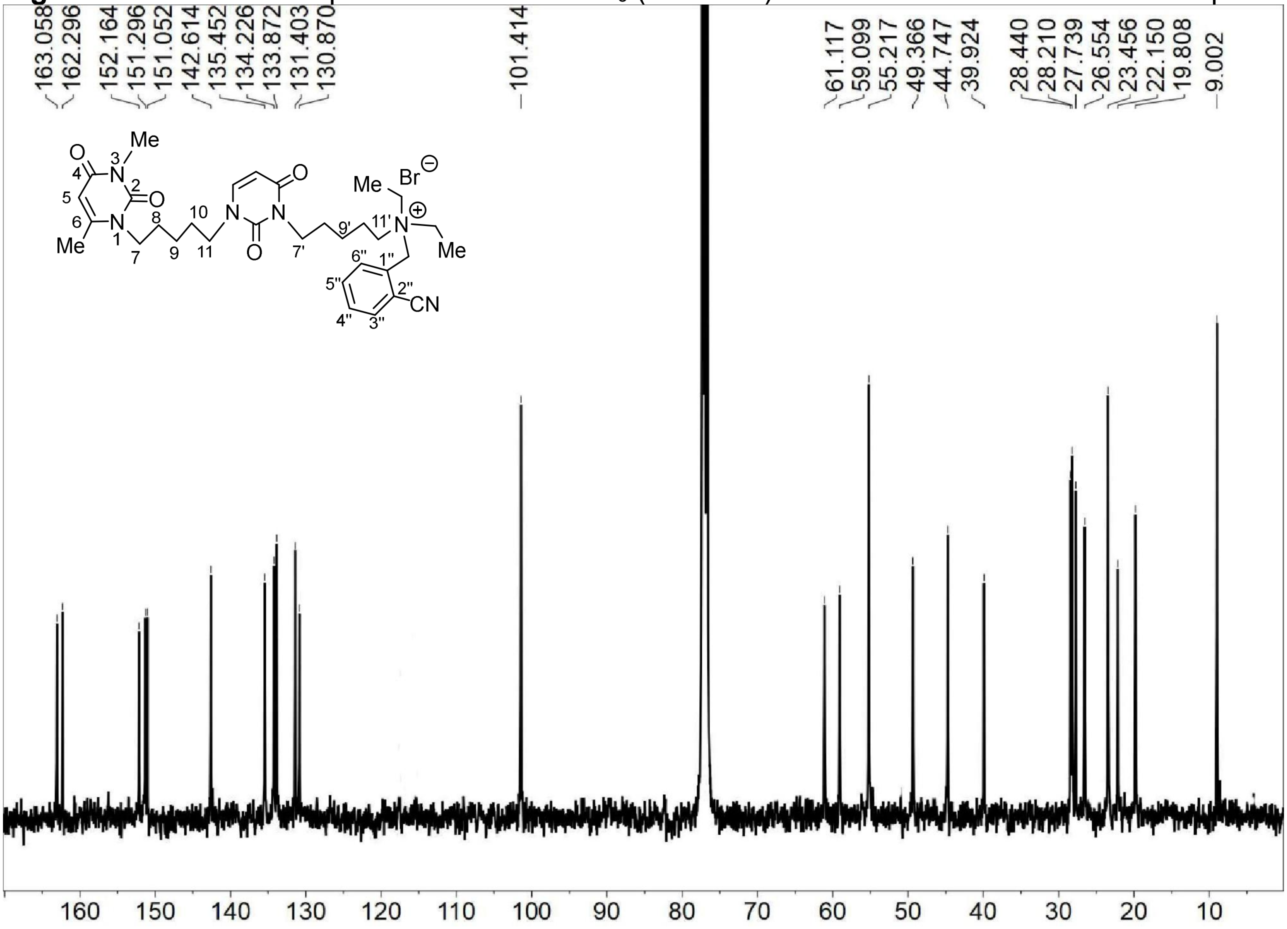

Figure S9. 1D ^{13}C NMR spectrum of **2c** in $CDCl_3$ (400 MHz) at T = 303 K. x - residual solvent peak.

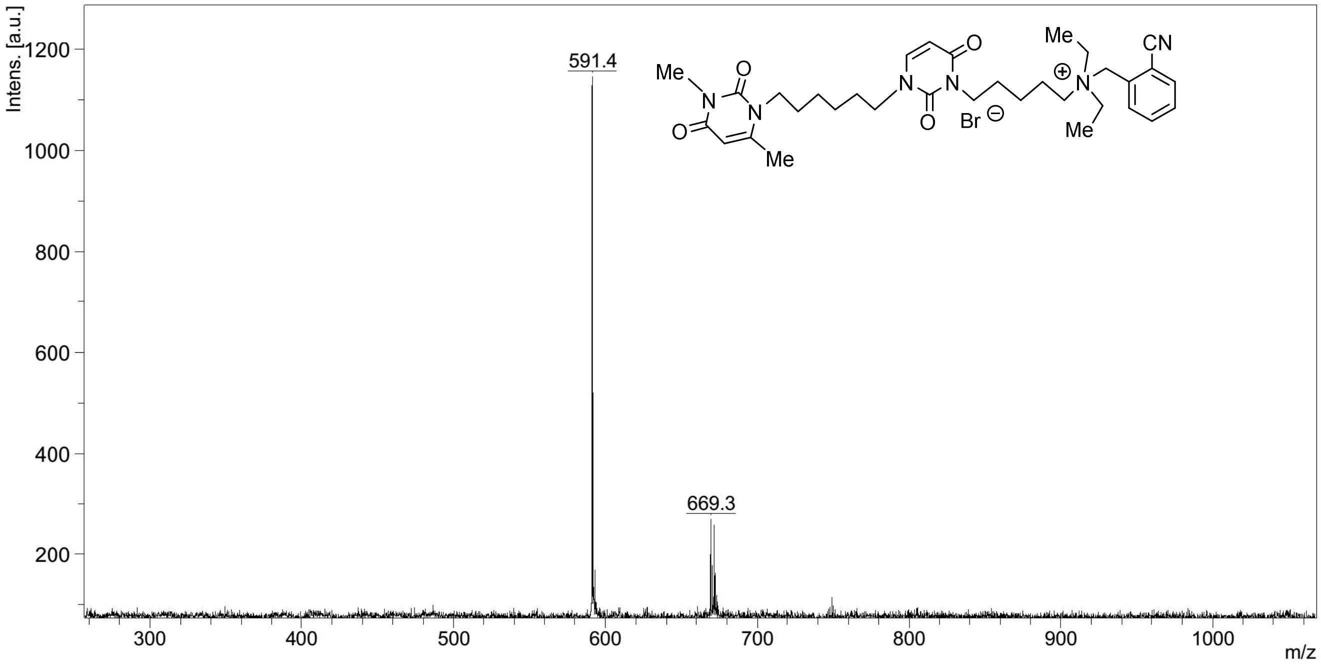

Figure S10. MALDI-TOF mass spectrum of **2d**.

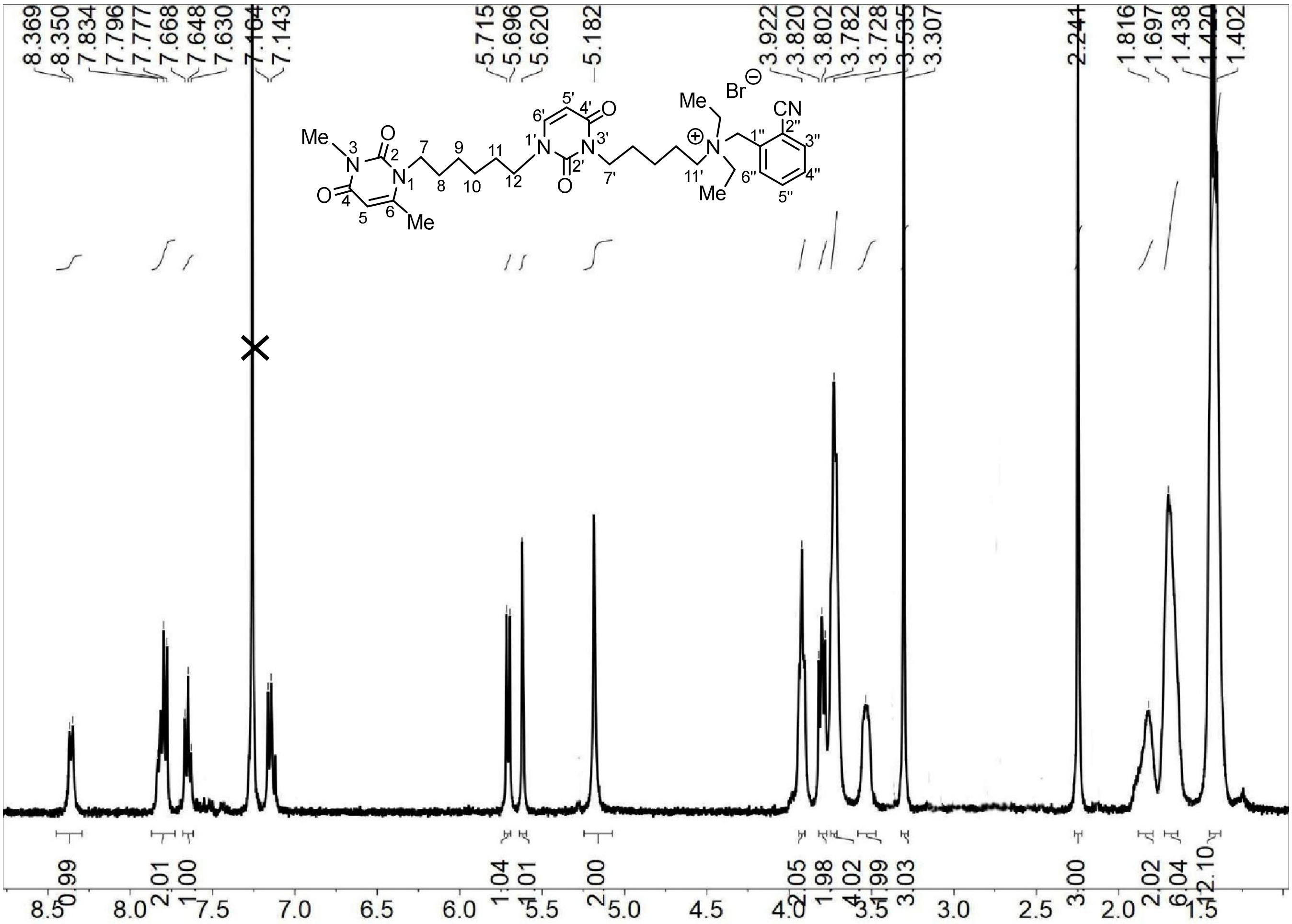

Figure S11. 1D ^{1}H NMR spectrum of **2d** in $CDCl_3$ (400 MHz) at T = 303 K. x - residual solvent peak.

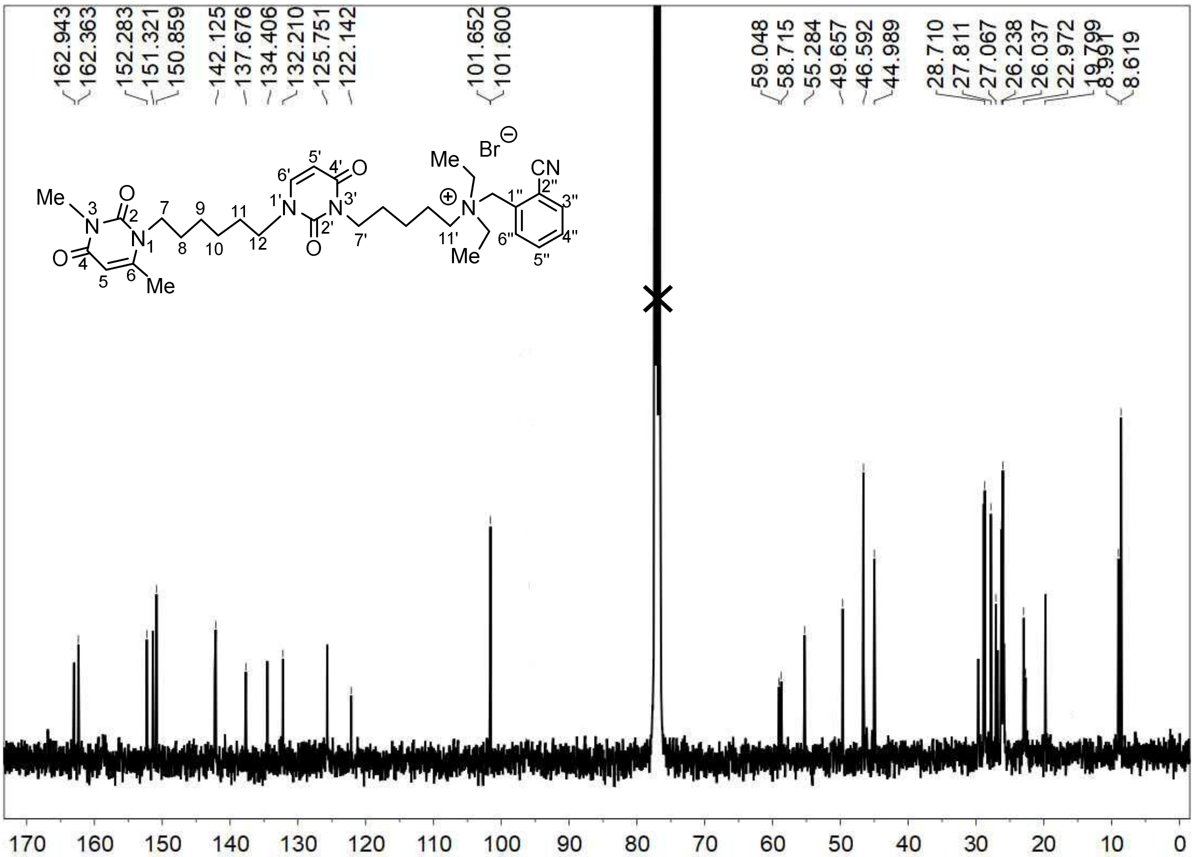

Figure S12. 1D ^{13}C NMR spectrum of **2d** in $CDCl_3$ (100 MHz) at T = 303 K. x – residual solvent peak.

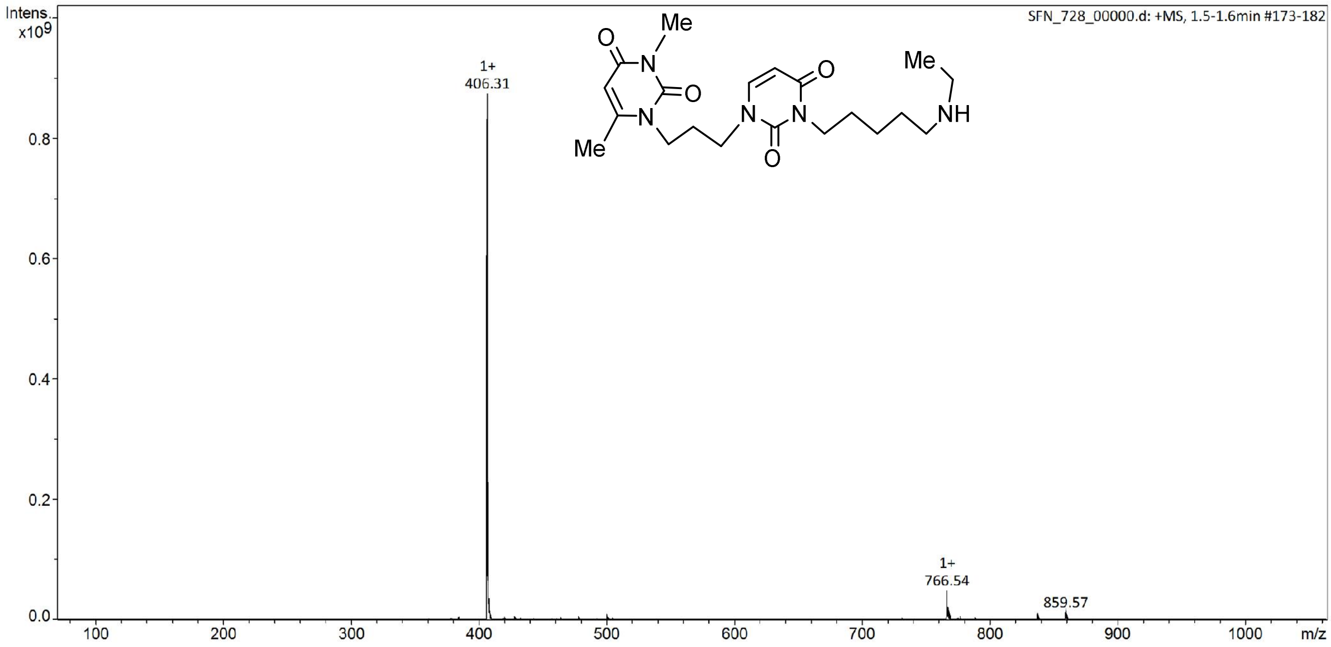

Figure S13. ESI mass spectrum of **10a**.

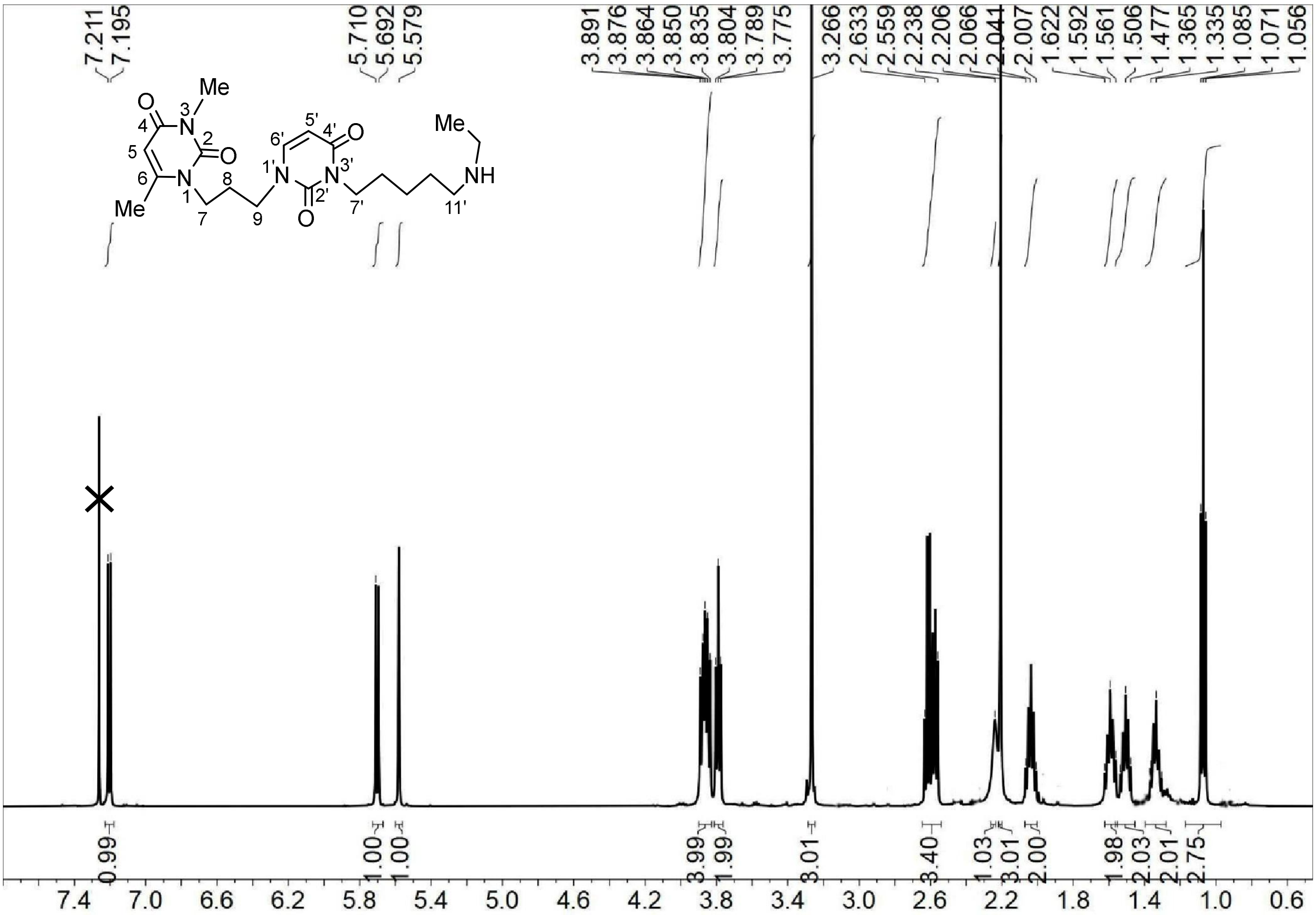

Figure S14. 1D ^{1}H NMR spectrum of **10a** in $CDCl_3$ (400 MHz) at T = 303 K. x – residual solvent peak.

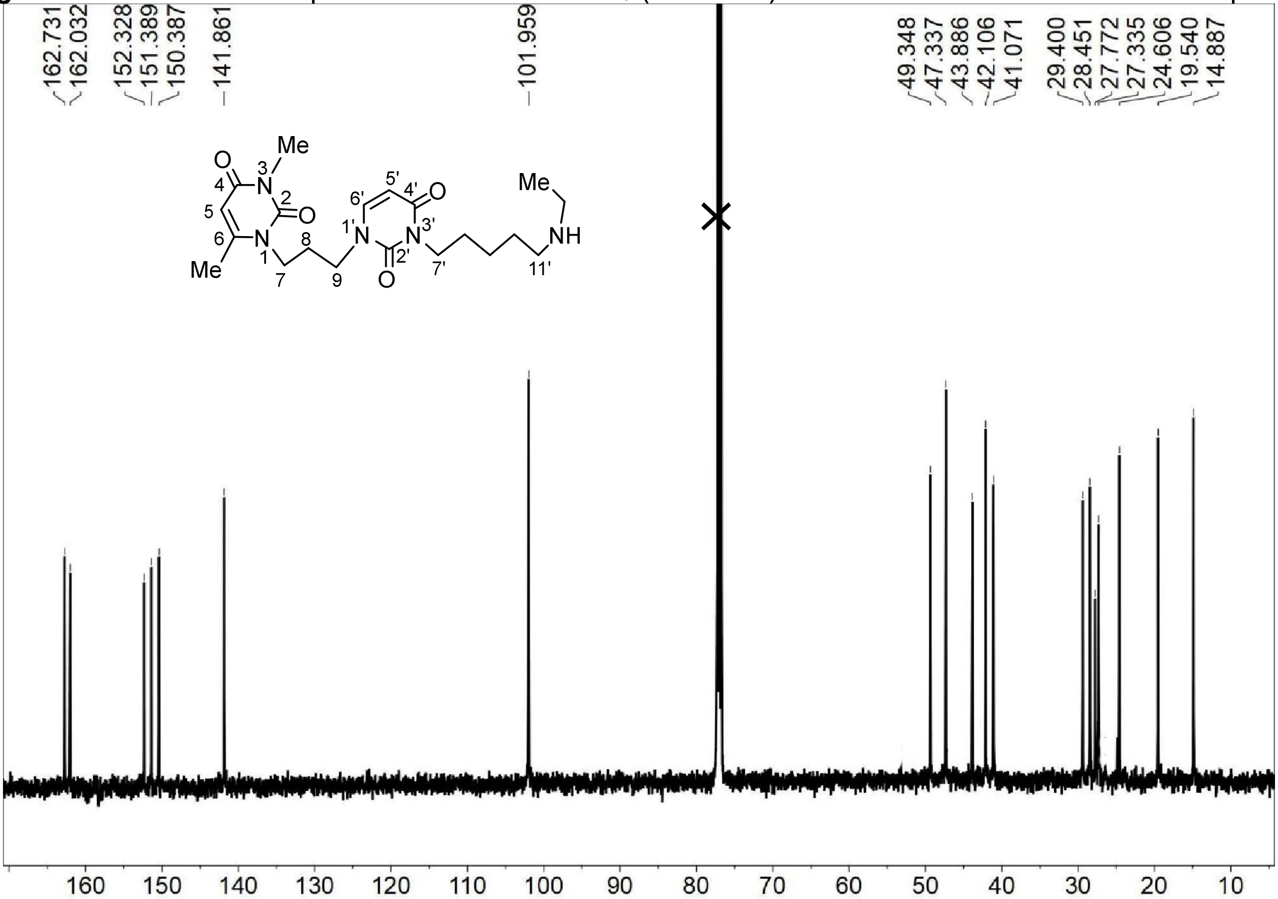

Figure S15. 1D ^{13}C NMR spectrum of **10a** in $CDCl_3$ (100 MHz) at T = 303 K. x - residual solvent peak.

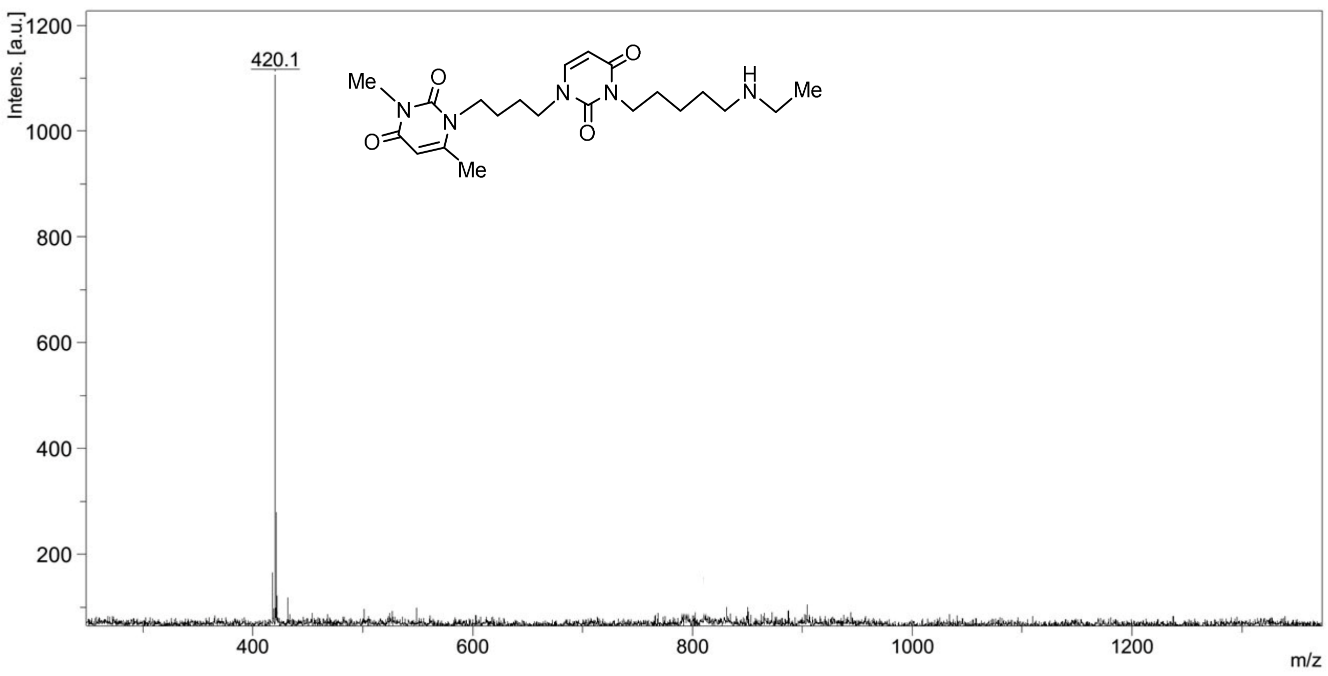

Figure S16. MALDI-TOF mass spectrum of **10b**.

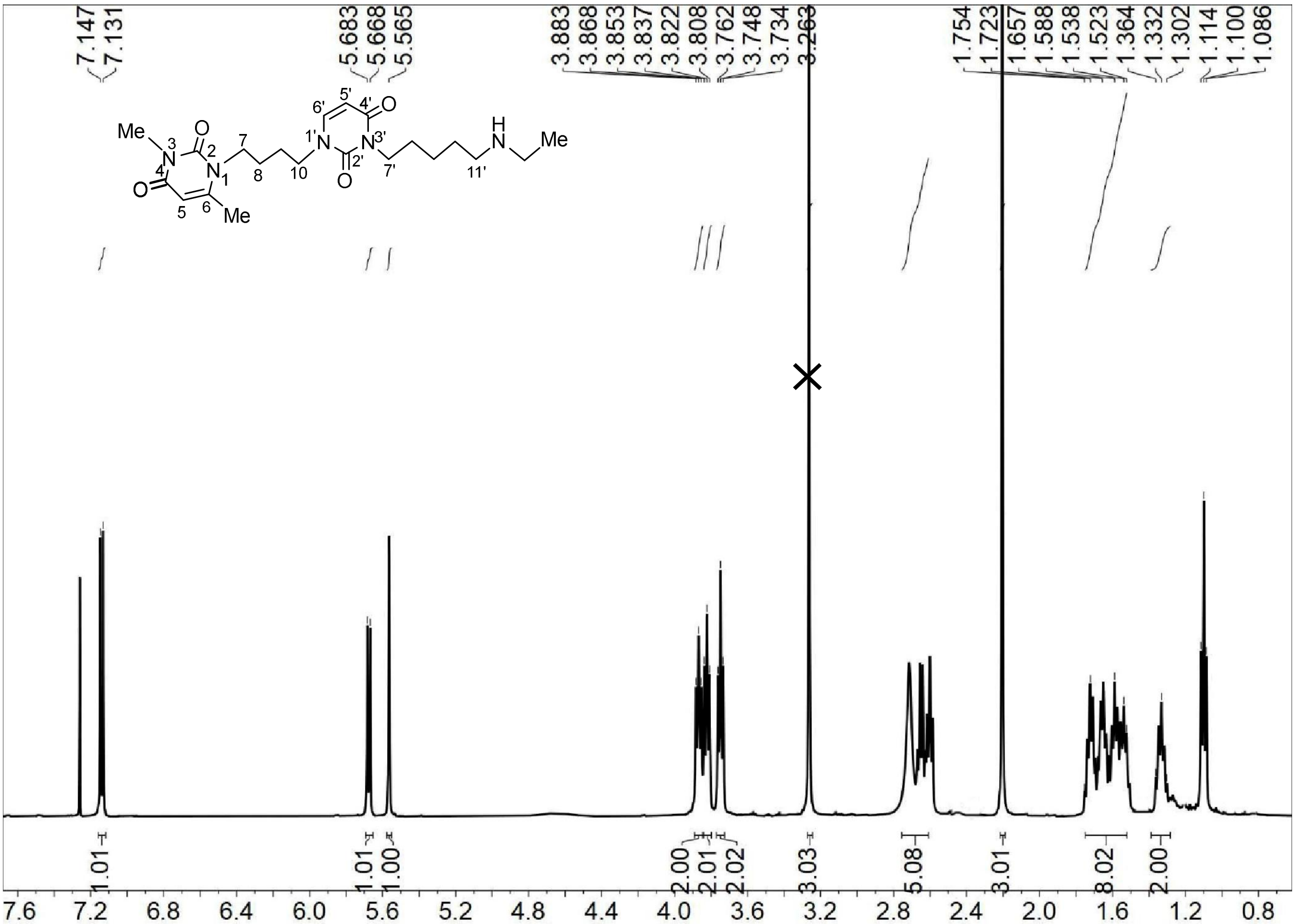

Figure S17. 1D ^{1}H NMR spectrum of **10b** in $CDCl_3$ (400 MHz) at T = 303 K. x – residual solvent peak.

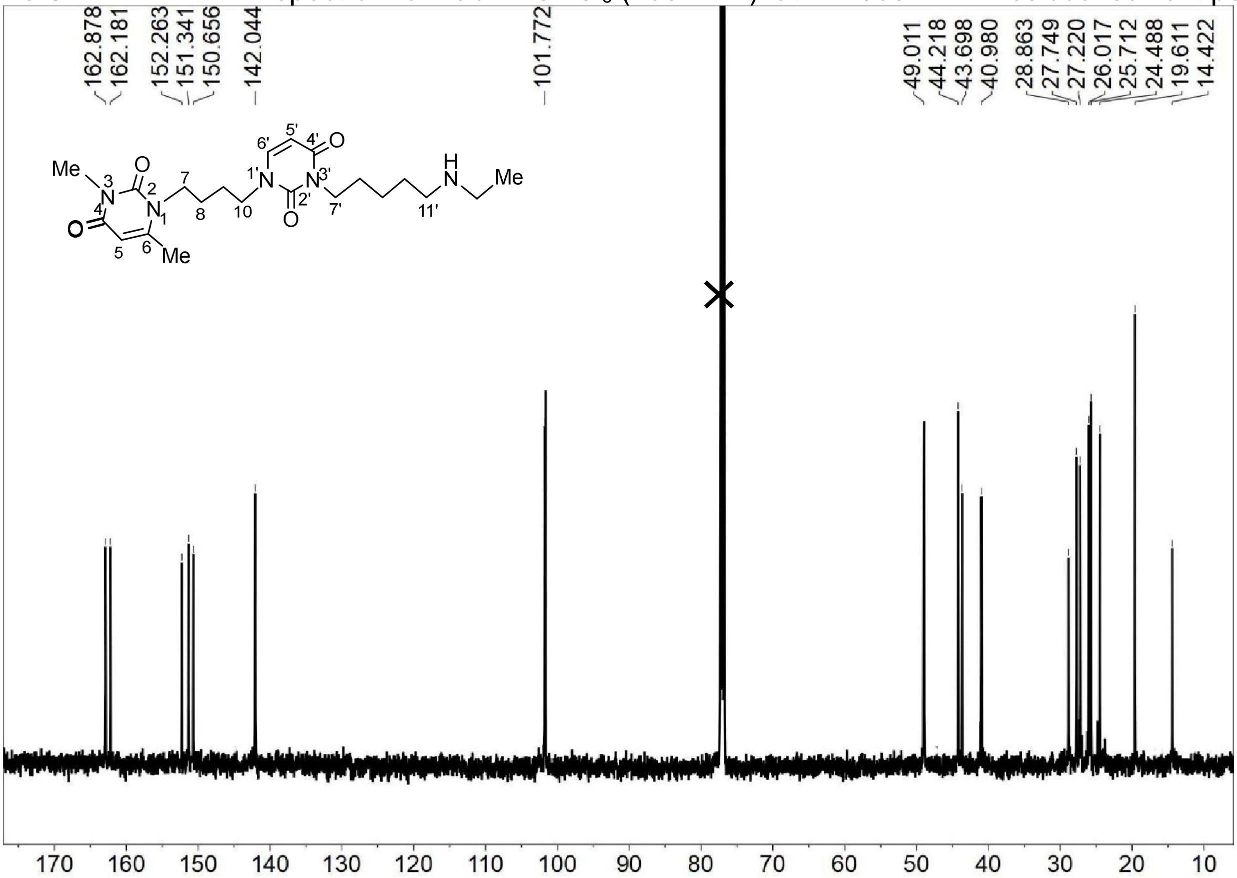

Figure S18. 1D ^{13}C NMR spectrum of **10b** in $CDCl_3$ (100 MHz) at T = 303 K. x - residual solvent peak.

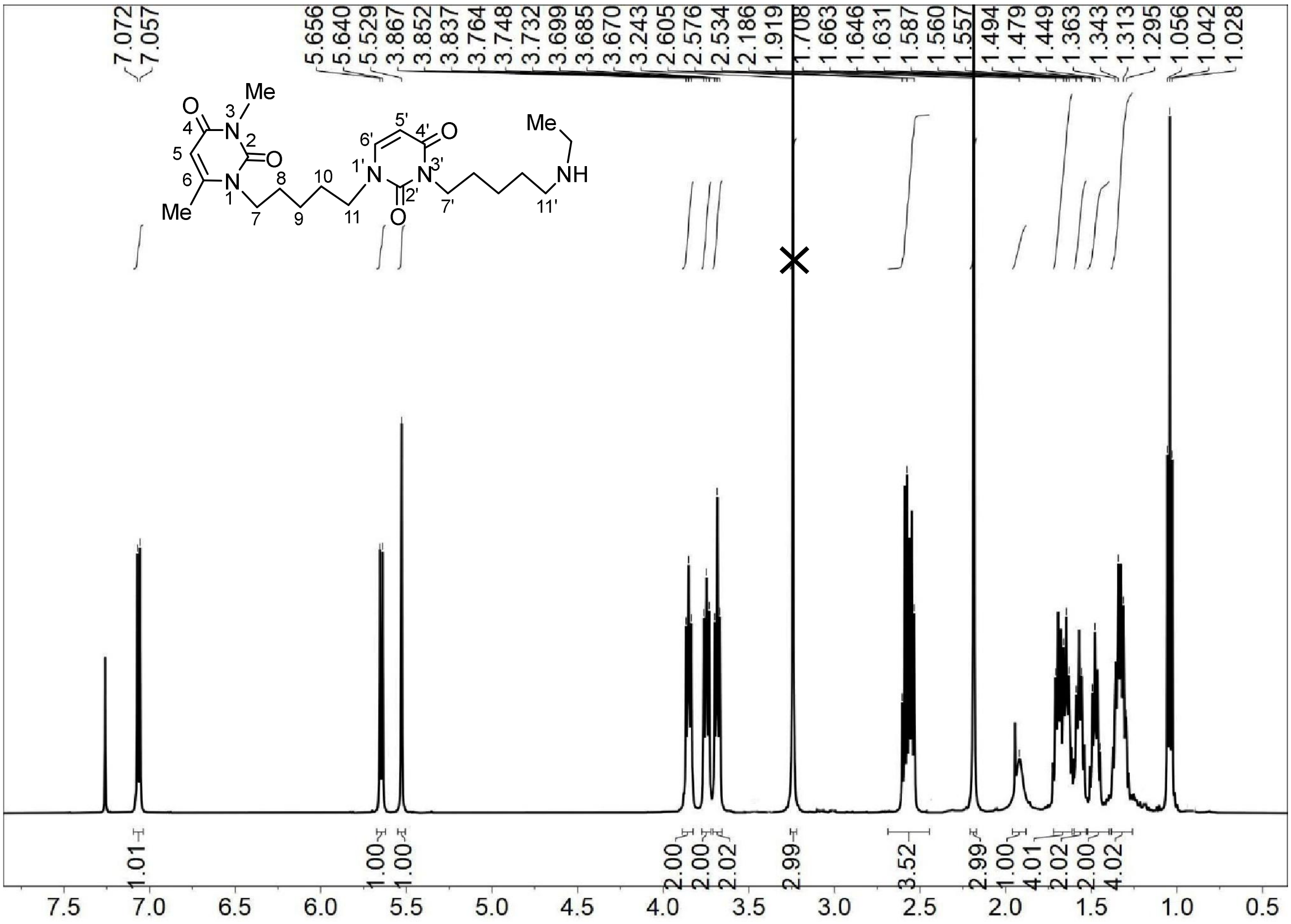

Figure S19. 1D ^{1}H NMR spectrum of **10c** in $CDCl_3$ (400 MHz) at T = 303 K. x – residual solvent peak.

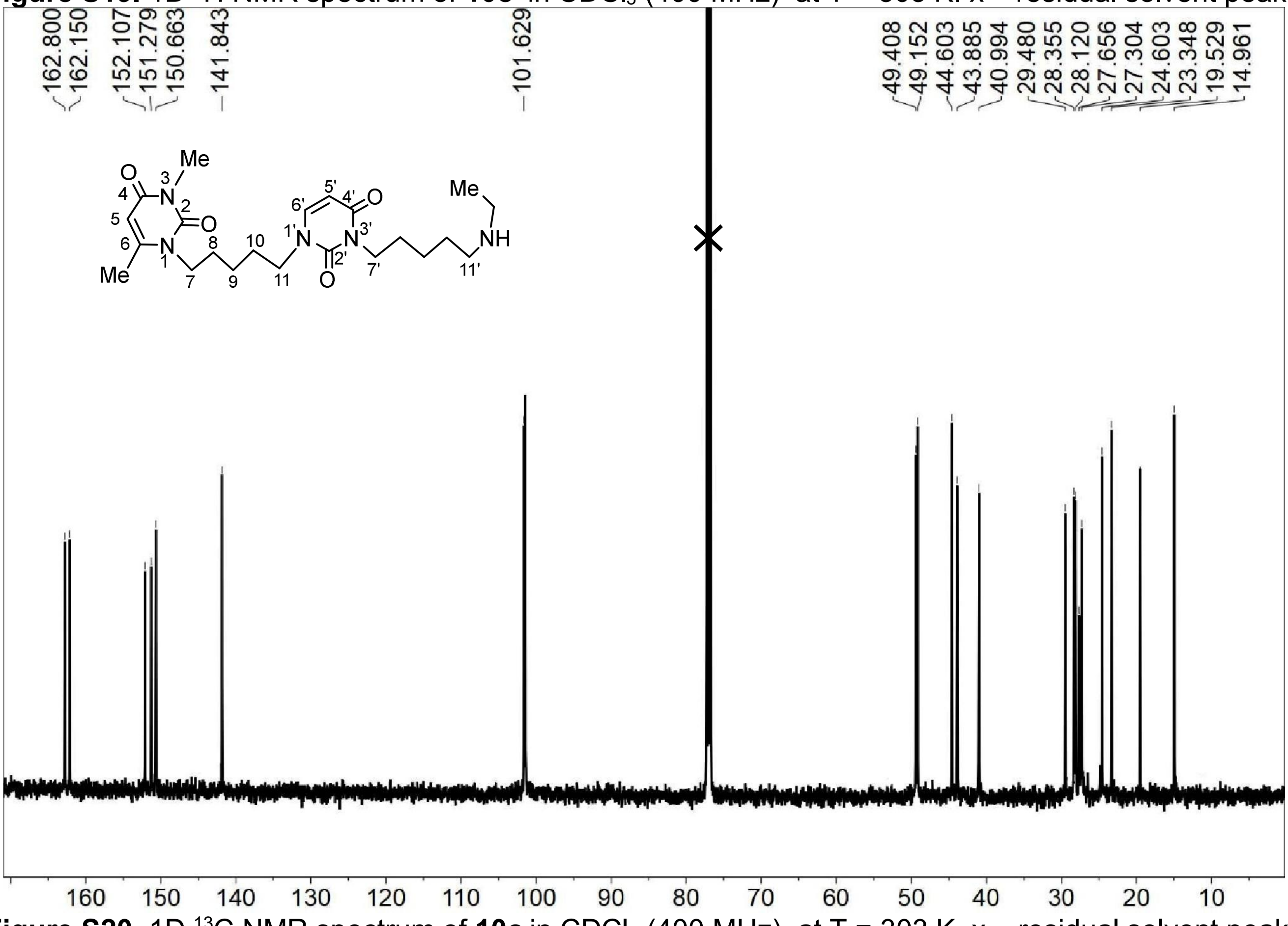

Figure S20. 1D ^{13}C NMR spectrum of **10c** in $CDCl_3$ (400 MHz) at T = 303 K. x – residual solvent peak.

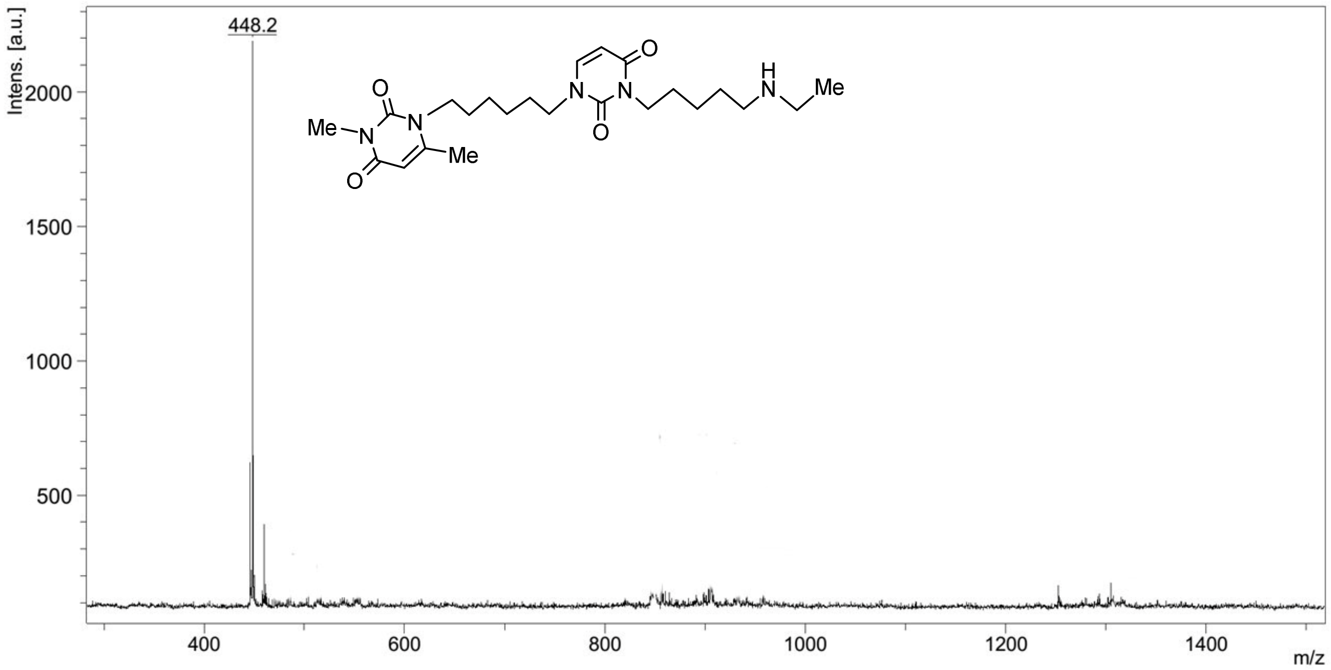

Figure S21. MALDI-TOF mass spectrum of **10d**.

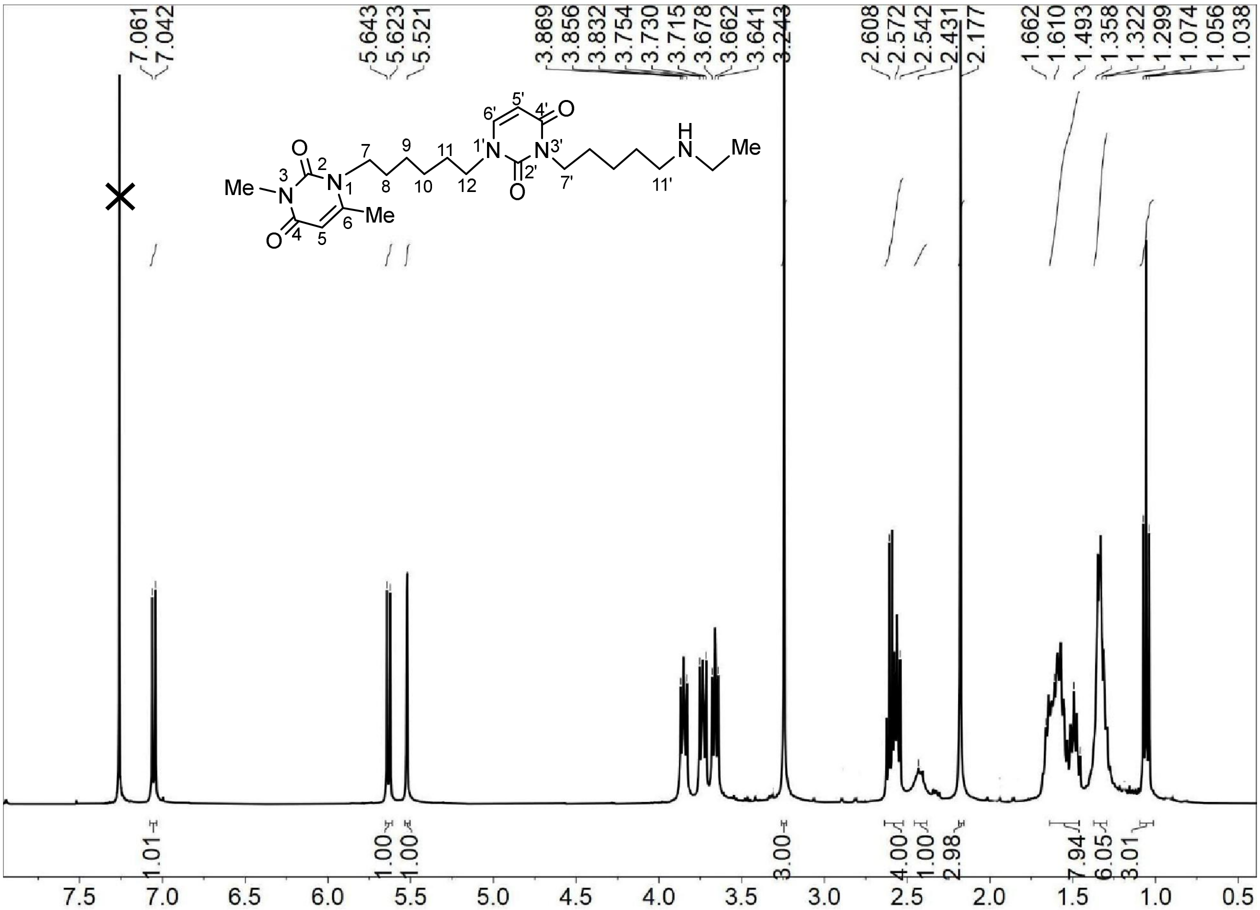

Figure S22. 1D ^{1}H NMR spectrum of **10d** in $CDCl_3$ (400 MHz) at T = 303 K. x – residual solvent peak.

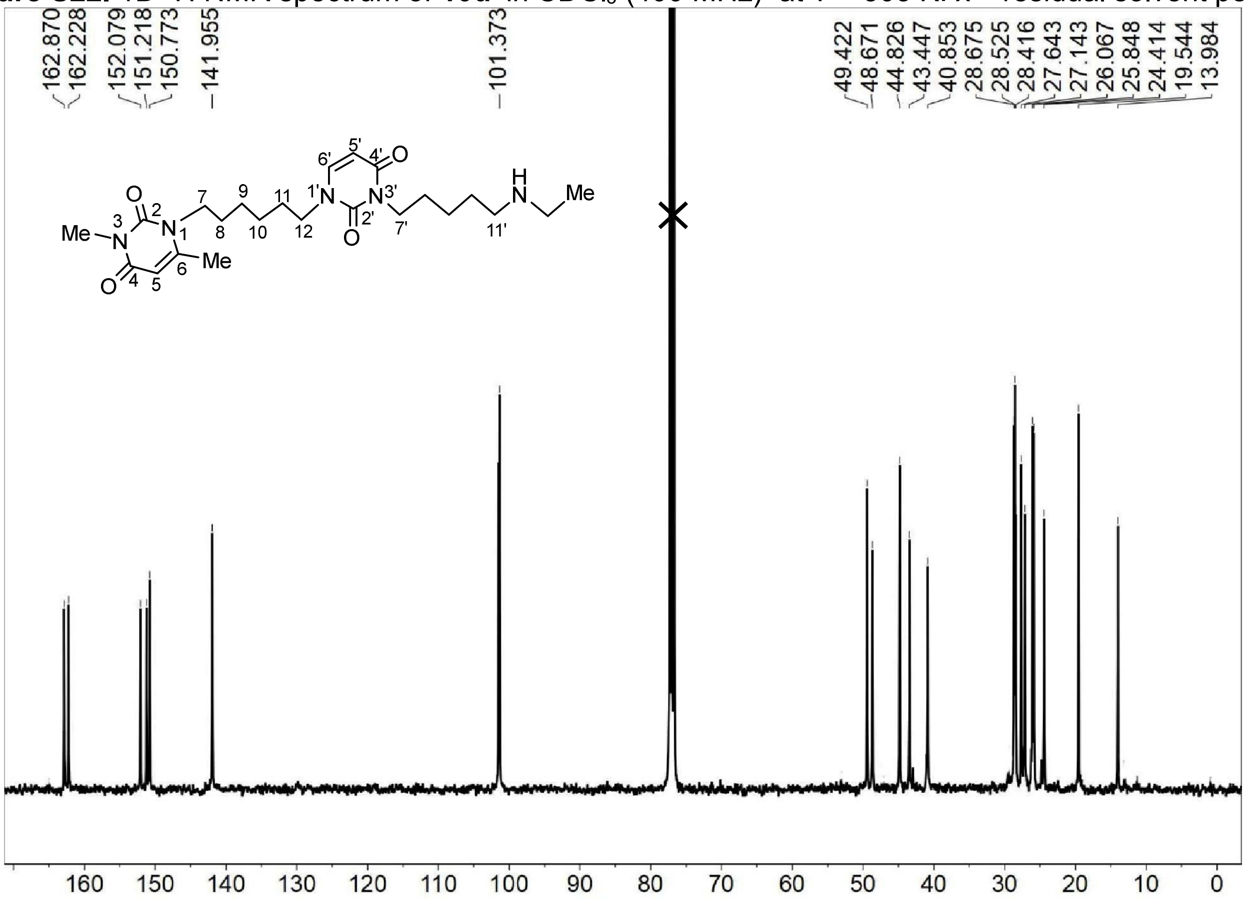

Figure S23. 1D ^{13}C NMR spectrum of **10d** in $CDCl_3$ (400 MHz) at T = 303 K. x – residual solvent peak.

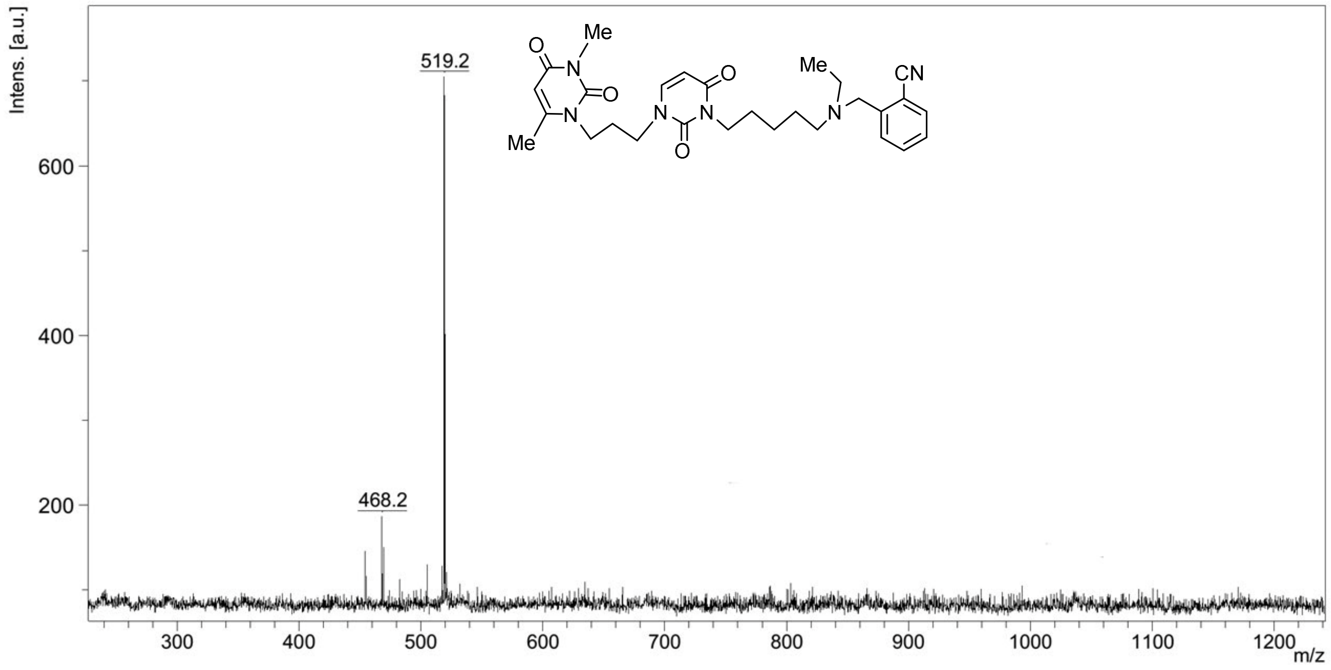

Figure S24. MALDI-TOF mass spectrum of **3a**.

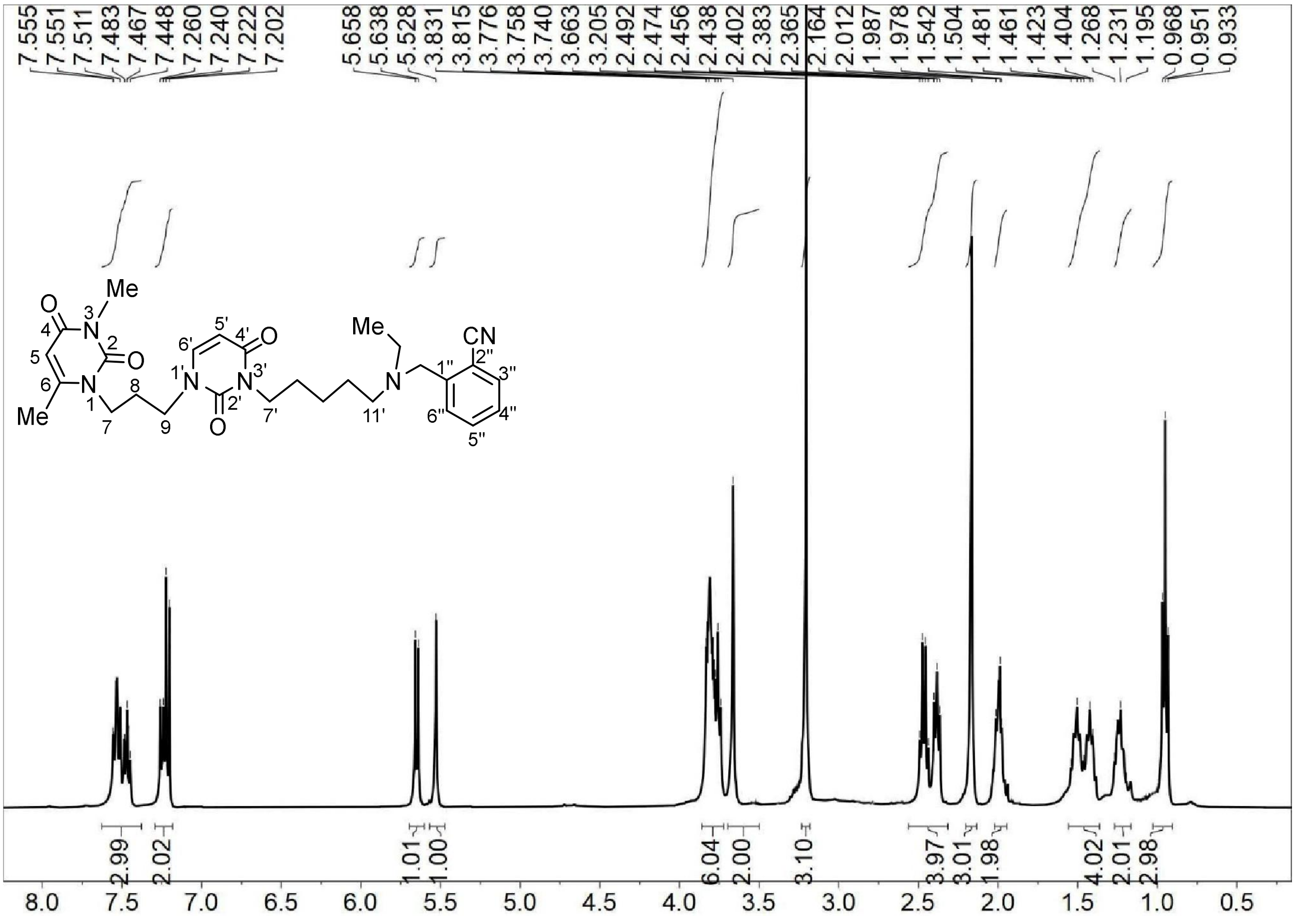

Figure S25. 1D ^{1}H NMR spectrum of **3a** in $CDCl_3$ (400 MHz) at T = 303 K. x – residual solvent peak.

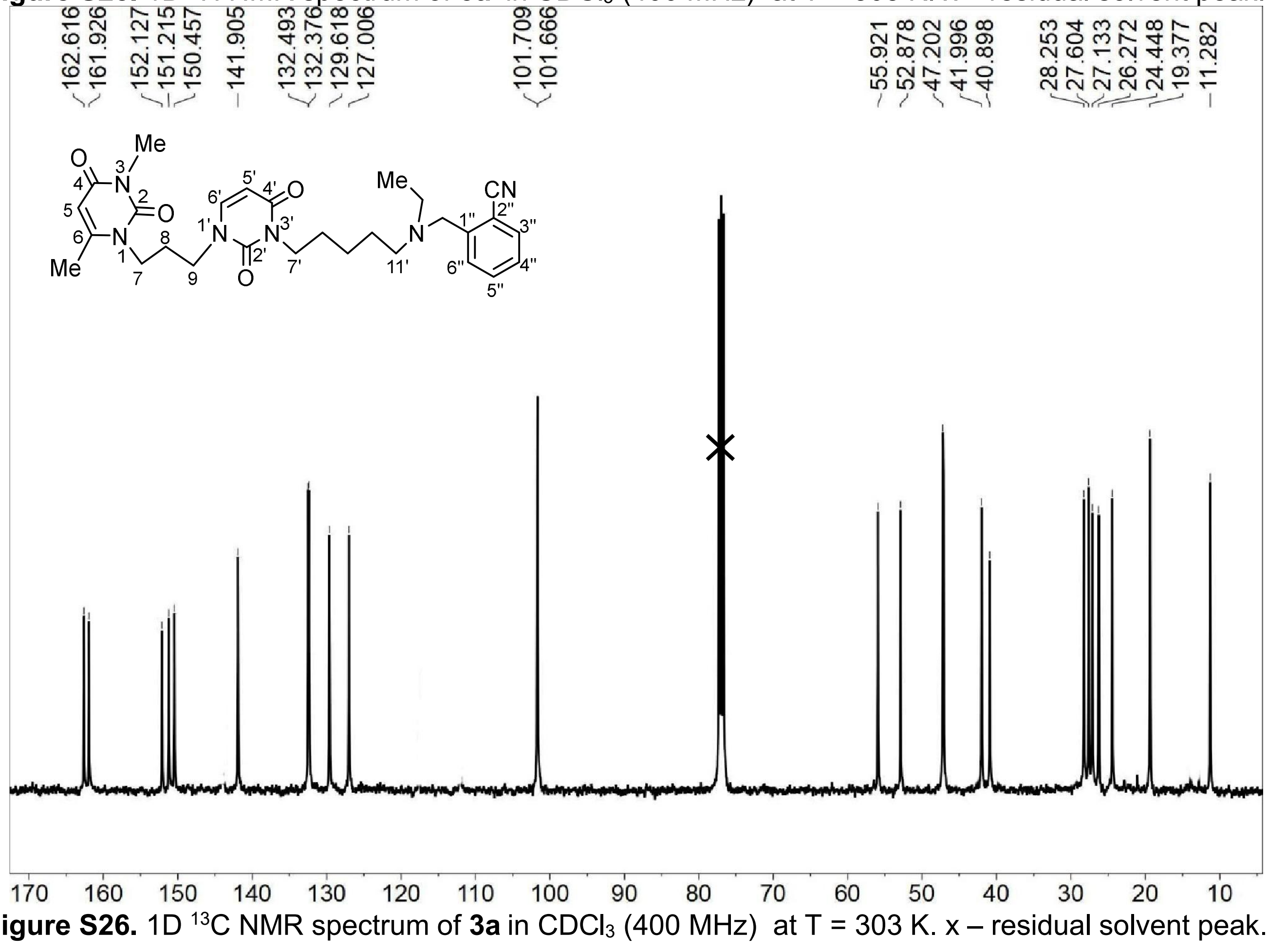

Figure S26. 1D ^{13}C NMR spectrum of **3a** in $CDCl_3$ (400 MHz) at T = 303 K. x – residual solvent peak.

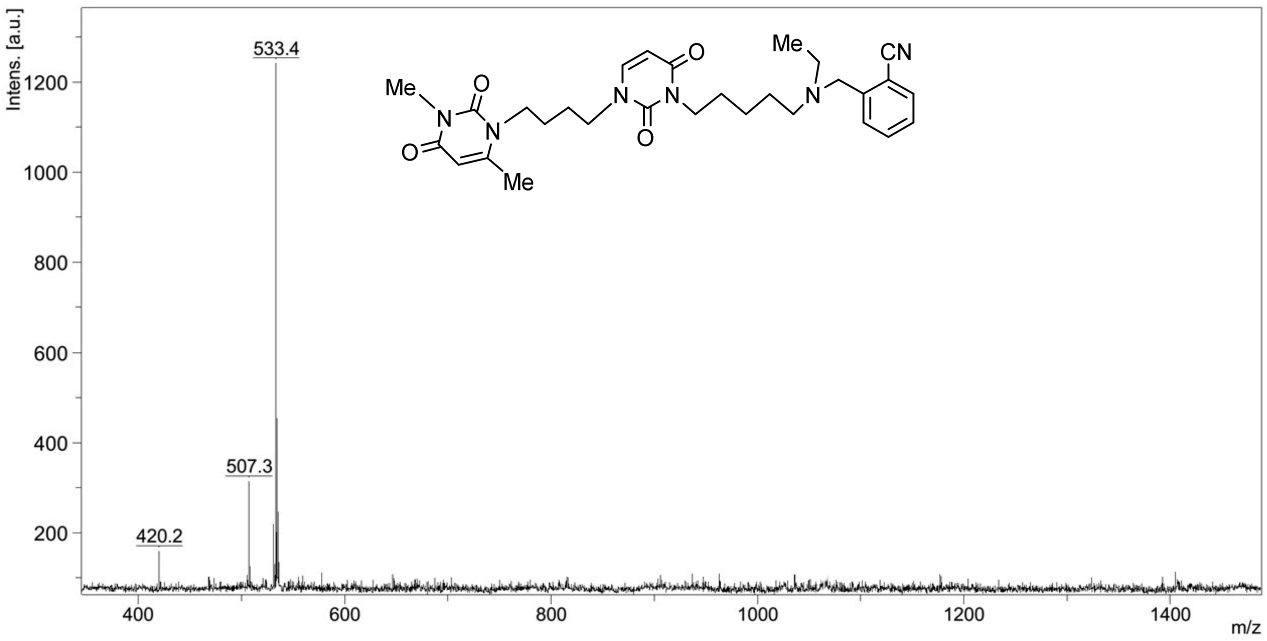

Figure S27. MALDI-TOF mass spectrum of **3b**.

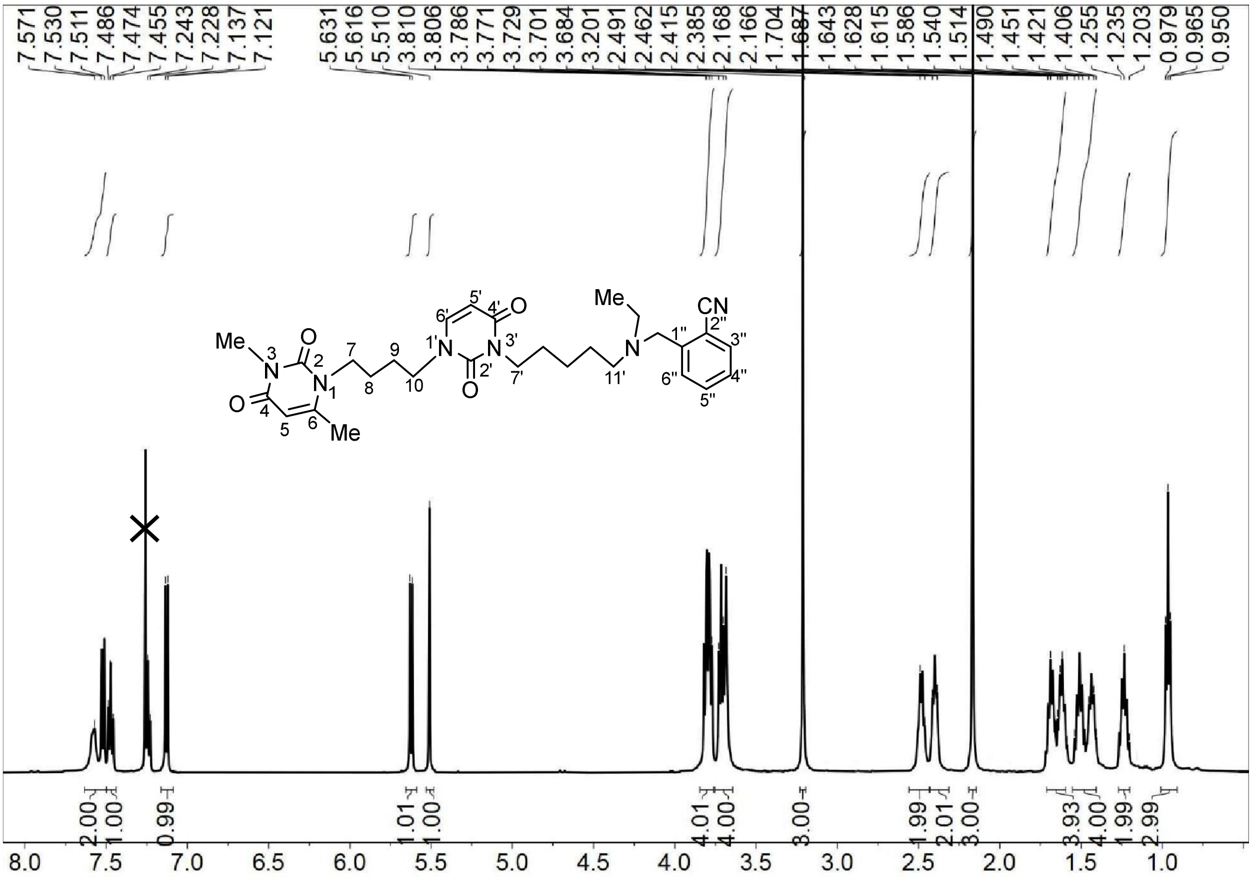

Figure S28. 1D ^{1}H NMR spectrum of **3b** in $CDCl_3$ (400 MHz) at T = 303 K. x – residual solvent peak.

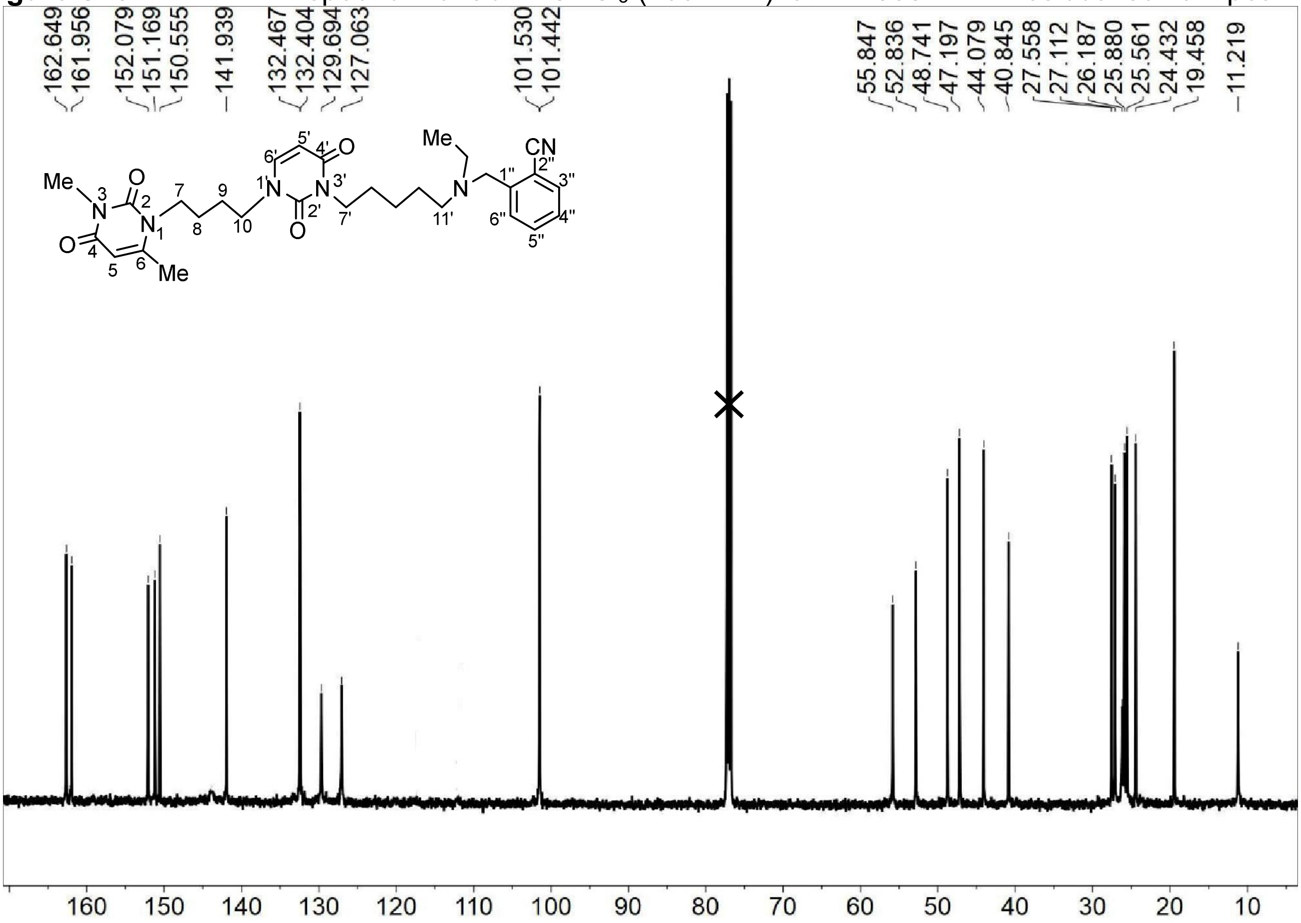

Figure S29. 1D ^{13}C NMR spectrum of **3b** in $CDCl_3$ (400 MHz) at T = 303 K. x – residual solvent peak.

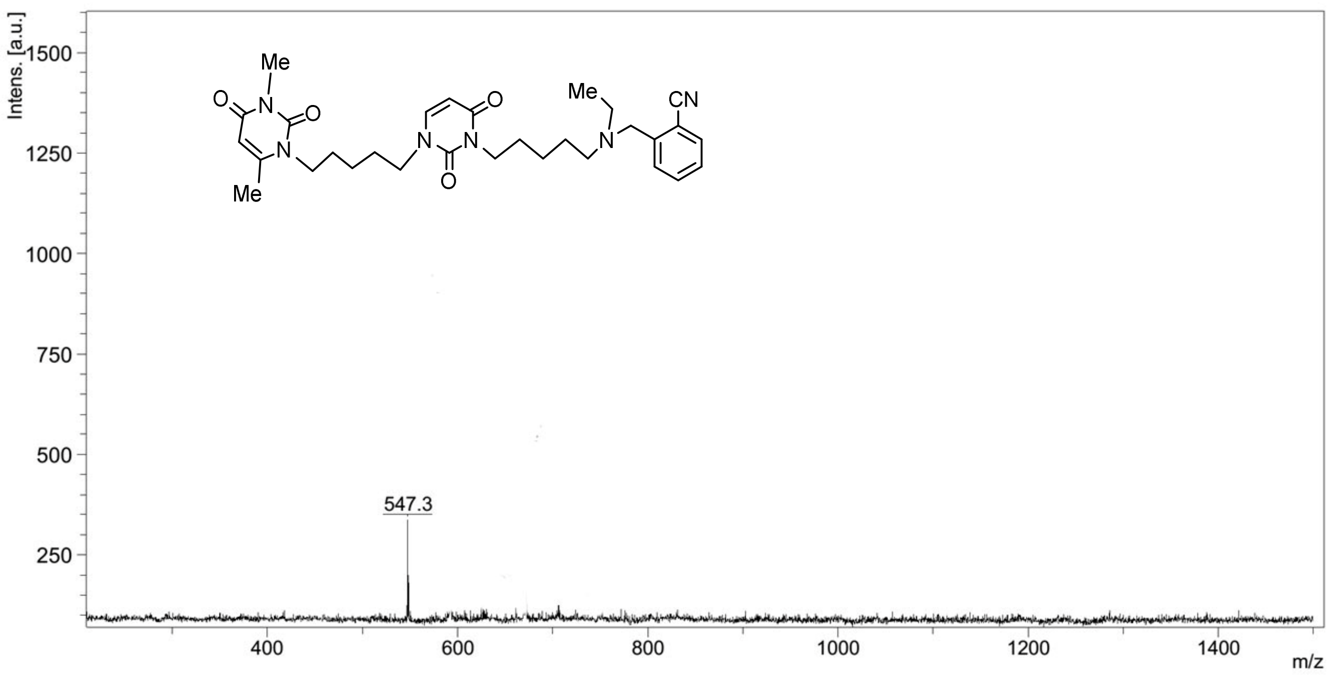

Figure S30. MALDI-TOF mass spectrum of **3c**.

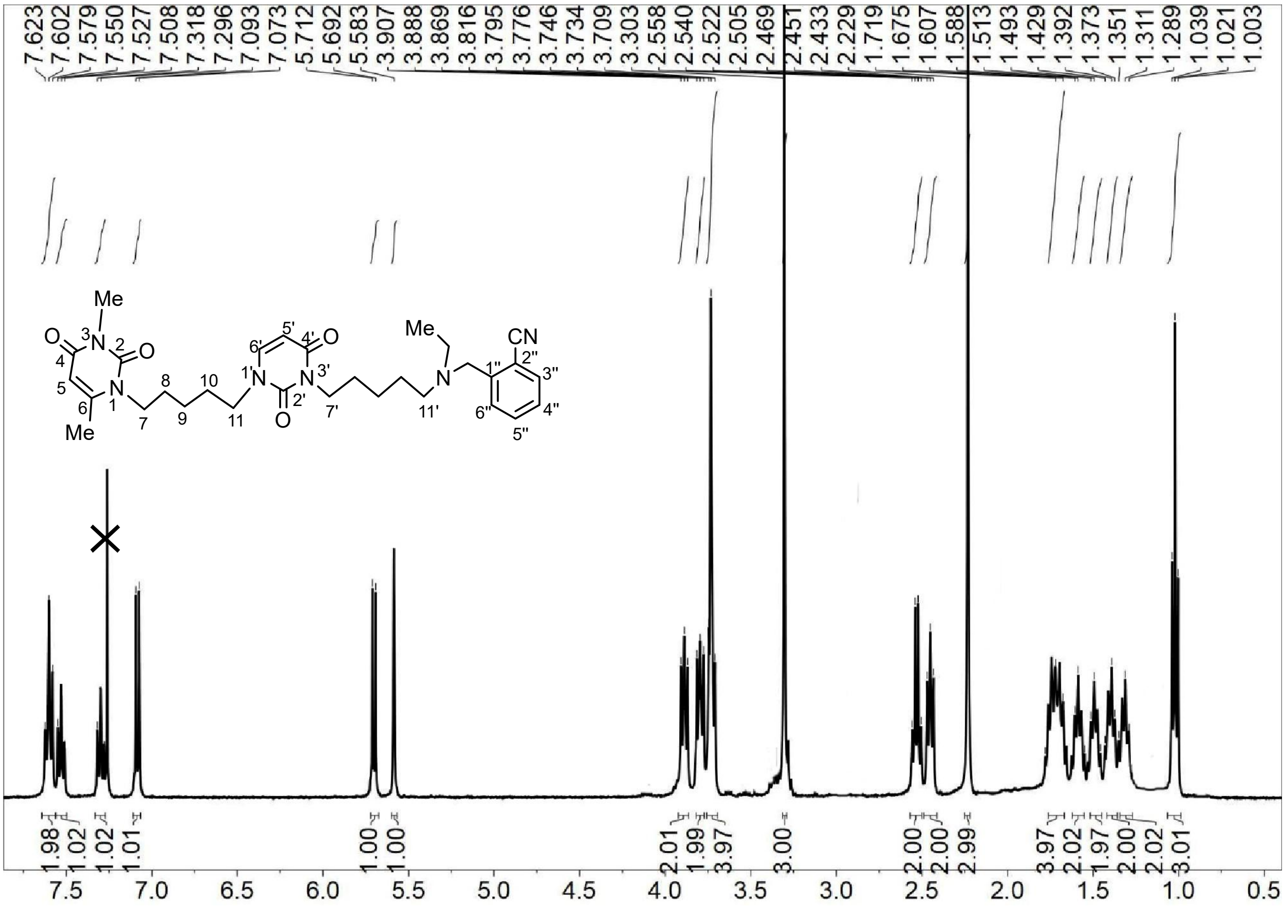

Figure S31. 1D ^{1}H NMR spectrum of **3c** in $CDCl_3$ (400 MHz) at T = 303 K. x – residual solvent peak.

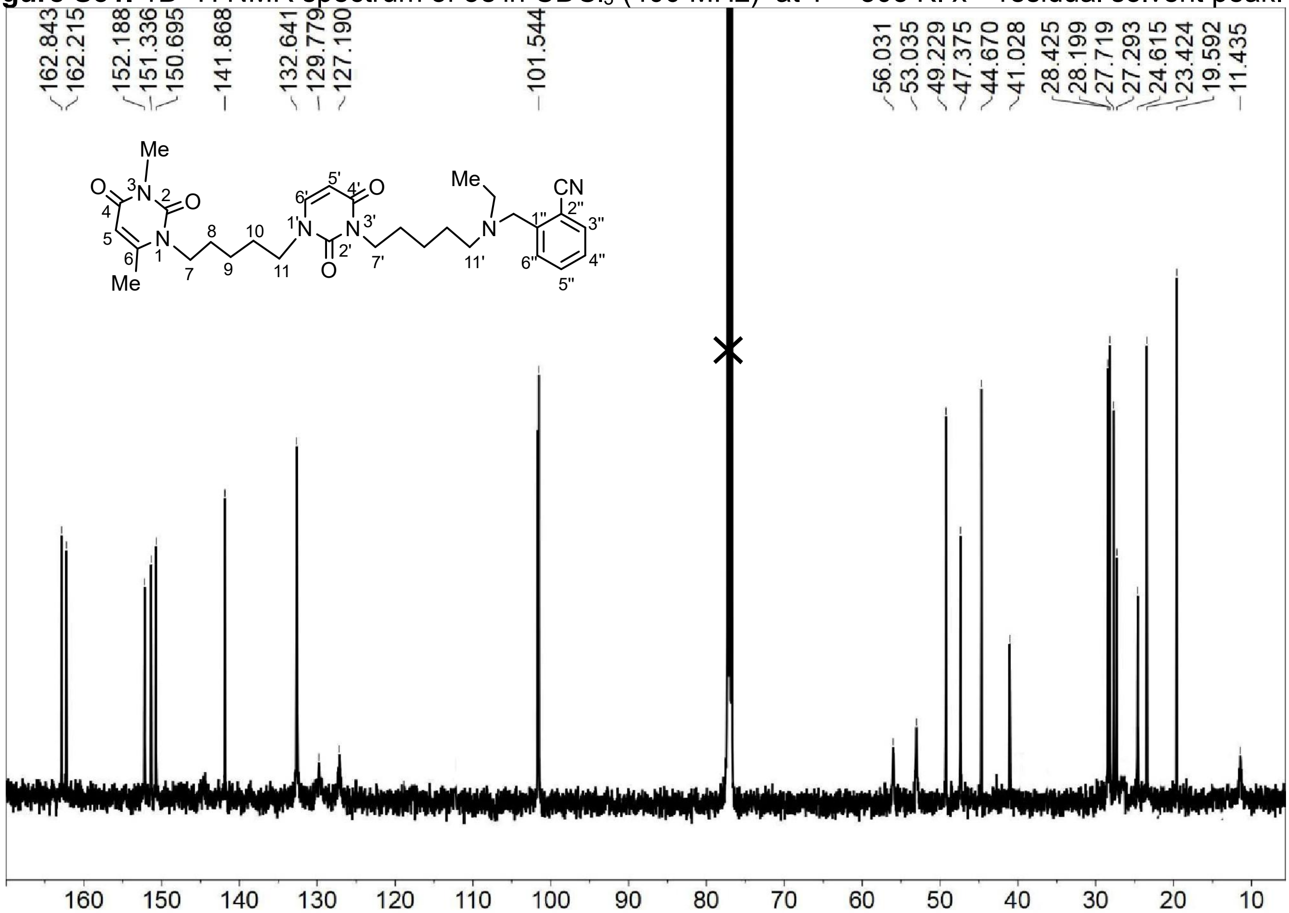

Figure S32. 1D ^{13}C NMR spectrum of **3c** in $CDCl_3$ (400 MHz) at T = 303 K. x – residual solvent peak.

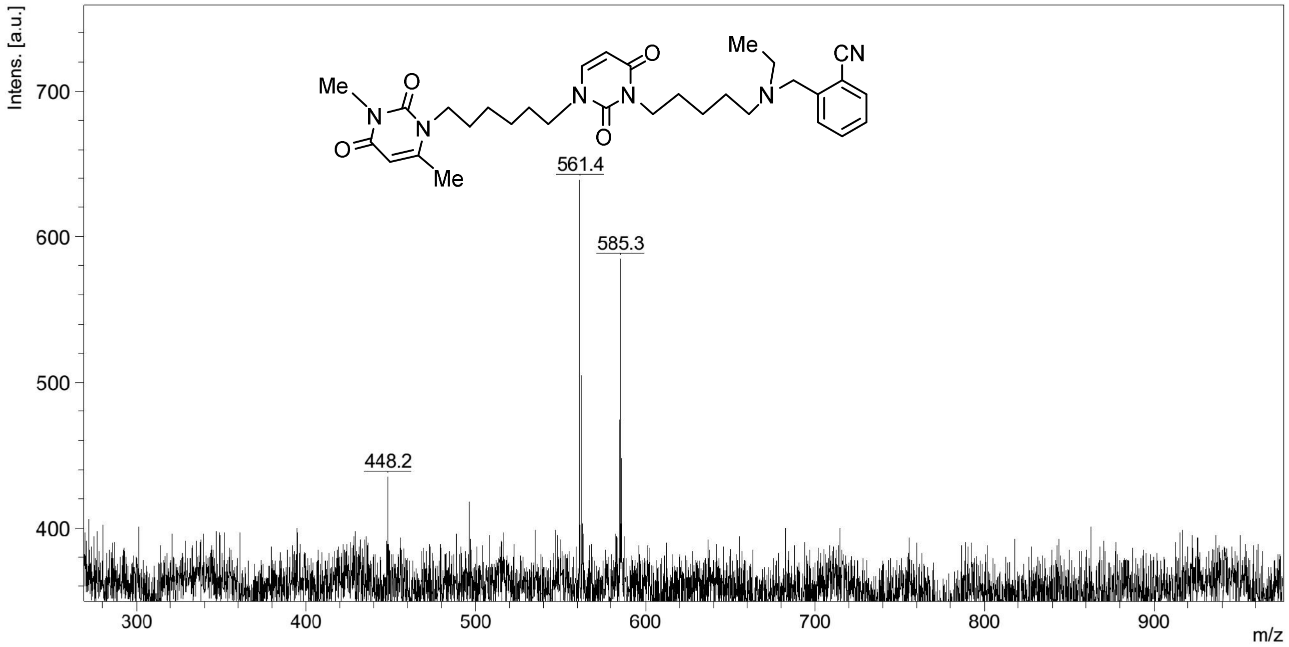

Figure S33. MALDI-TOF mass spectrum of **3d**.

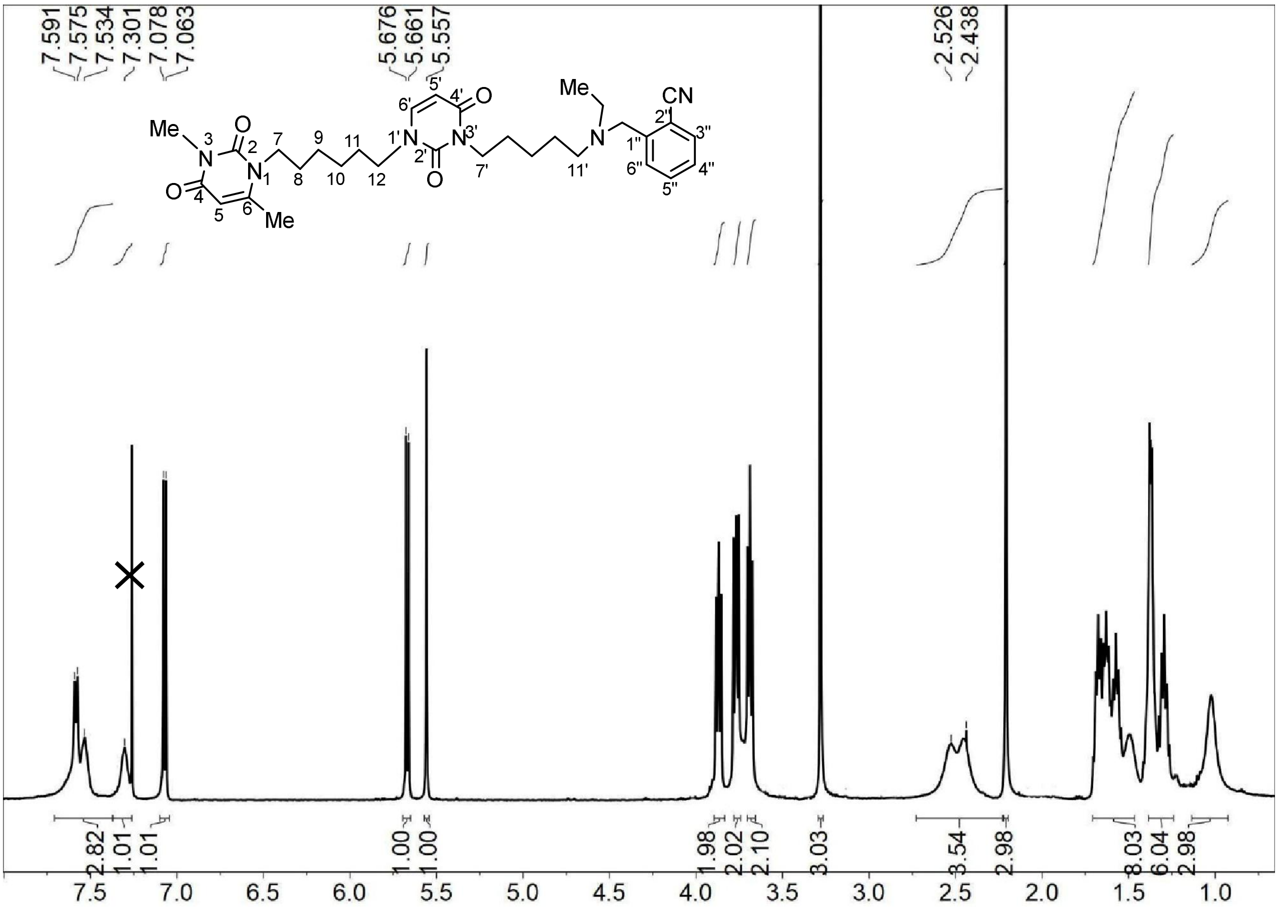

Figure S34. 1D ^{1}H NMR spectrum of **3d** in $CDCl_3$ (400 MHz) at T = 303 K. x – residual solvent peak.

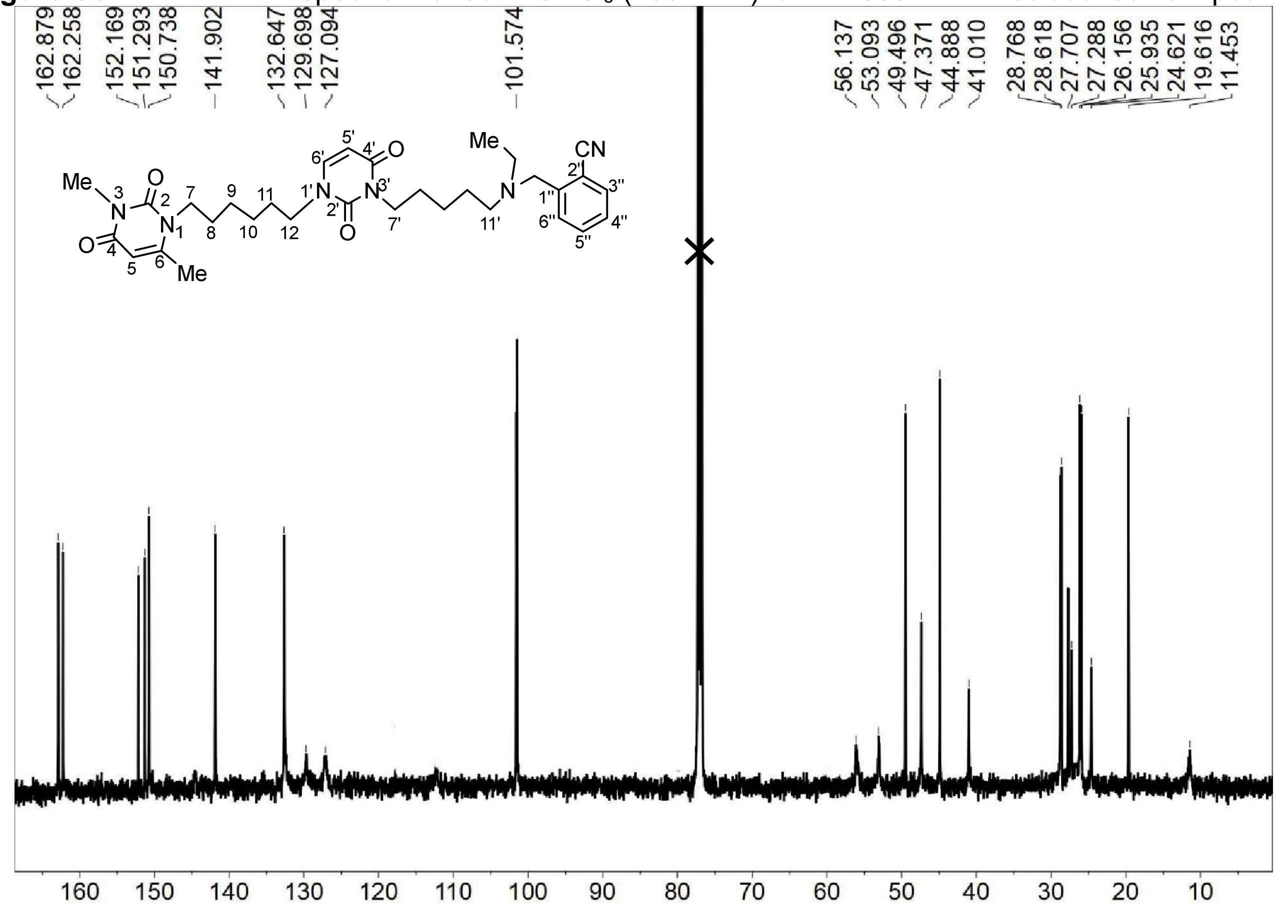

Figure S35. 1D ^{13}C NMR spectrum of **3d** in $CDCl_3$ (400 MHz) at T = 303 K. x – residual solvent peak.
